# Carbon Quantum Dots: From Mechanisms to Biomedical Applications

**DOI:** 10.1002/mco2.71062

**Published:** 2026-10-13

**Authors:** Bingxin Fan, Nianqiang Jin, Nuo Jin, Jiayu Shen, Xinliang Duan, Sichen Han, Yao Yuan, Zilin Wang

**Affiliations:** ^1^ Department of Oral and Maxillofacial Surgery Jilin Provincial Key Laboratory of Tooth Development and Bone Remodeling School and Hospital of Stomatology Jilin University Changchun China; ^2^ School of Stomatology Stomatological Hospital Southern Medical University Guangzhou People's Republic of China

**Keywords:** carbon quantum dots, tissue regeneration, regulatory mechanisms, precision therapy

## Abstract

Carbon quantum dots (CQDs) have emerged as promising nanomaterials for biomedical applications, including bioimaging, drug delivery, biosensing, and tissue regeneration, owing to their favorable biocompatibility, tunable physicochemical properties, and diverse biological activities. Despite accumulating evidence of their regenerative potential, the mechanisms by which CQDs regulate complex tissue regeneration remain insufficiently integrated. In this Review, we synthesize recent advances in the biomedical applications of CQDs, with particular emphasis on material selection for specific disease contexts and their direct and indirect regulatory roles in tissue regeneration. We examine how CQDs modulate cellular functions, angiogenesis, stem cell differentiation, the immune microenvironment, and redox homeostasis through molecular pathways, and how their physicochemical properties shape these biological effects and regenerative outcomes. We further discuss emerging strategies for functional optimization, including multifunctional and stimuli‐responsive CQD systems. Finally, we critically assess key translational barriers, including structural characterization, biological evaluation, safety assessment, material standardization, and manufacturing consistency. By linking CQDs’ properties to molecular mechanisms and regenerative outcomes, this Review provides a conceptual framework for the rational design of CQDs and the development of more effective regenerative therapies.

## Graphical abstract

Carbon quantum dots (CQDs) are promising carbon‐based nanomaterials with broad biomedical applications, including bioimaging, drug delivery, biosensing, and tissue regeneration. This Review summarizes the biological mechanisms of CQDs in tissue regeneration, focusing on their regulation of cellular functions, immune responses, and redox balance. We also discuss their applications in precision medicine, material design principles, and challenges for clinical translation.

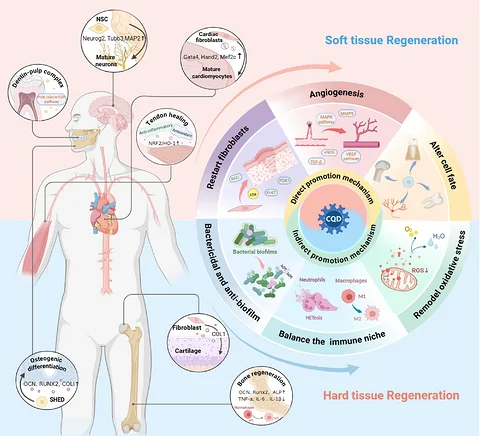

## 1 Introduction

The rapid development of nanomaterials has expanded their applications in biomedicine and provided new strategies for precision therapy and tissue repair and regeneration. Among these materials, carbon quantum dots (CQDs) have emerged as promising nanomaterials due to their outstanding physicochemical properties. CQDs were first reported by Xu et al. [1] and have since attracted increasing attention in biomedical research. Owing to their excellent biocompatibility, unique optical properties, stable physicochemical characteristics, and abundant surface modification sites, CQDs show great potential in various biomedical applications, including precision therapy and tissue regeneration [2, 3]. Recent studies have demonstrated that CQDs can serve not only as sensitive platforms for biological signal detection and efficient carriers for drug delivery, but also as active regulators of biological processes. Their efficient transmembrane transport and uniform distribution within subcellular compartments provide unique advantages for regulating tissue regeneration in a spatial and temporal manner. In addition, the abundant functional groups on the surface of CQDs and their enzyme‐like activities enable precise and efficient regulation of intracellular signaling pathways and biological reactions [4, 5]. Therefore, by activating endogenous repair mechanisms, CQDs may directly modulate tissue regeneration processes and have achieved promising therapeutic outcomes.

In this context, CQDs have gradually evolved from conventional imaging probes, delivery carriers, and sensing elements into emerging functional materials capable of actively regulating biological processes. Among their biomedical applications, tissue regeneration has received increasing attention due to its relatively well‐established research foundation and strong potential for clinical translation. As a highly complex and precisely regulated system, the regeneration process does not rely solely on the activation or migration of individual cells. Instead, it involves coordinated interactions among various cell types, in which cells regulate themselves or neighboring cells through autocrine and paracrine signaling to remodel the tissue microenvironment [6, 7]. This process is driven by dynamic interactions among cells, extracellular matrix components, cytokines, and growth factors that occur in a precisely controlled spatial and temporal manner [8, 9]. Activating the intrinsic repair mechanisms of the body is a key driving force for shifting this balance toward regeneration [10, 11]. The unique regulatory effects of CQDs on tissue cells, together with their remarkable antibacterial and anti‐inflammatory properties, have introduced new opportunities for regenerative medicine [9, 12]. However, current reviews of the biomedical applications of CQDs have mainly focused on their roles in cancer therapy and antibacterial strategies, while a systematic understanding of their applications and underlying mechanisms in tissue regeneration remains limited. Identifying specific biological processes and uncovering potential therapeutic targets are essential steps for advancing the clinical translation of biomaterials. Moreover, understanding the relationship between biomaterials and therapeutic targets provides critical guidance for the development of future therapeutic strategies.

This review summarizes the recent advances of CQDs in the biomedical field, covering a wide range of studies published over the past five years on precision therapy and tissue repair and regeneration. Starting from the fundamental properties of CQDs, this review provides an overview of their current applications and potential regulatory patterns in tissue regeneration, as well as in precision biomedical applications including bioimaging, drug delivery, and biosensing. We further discuss the direct and indirect mechanisms through which CQDs regulate tissue regeneration and describe the underlying molecular mechanisms, with particular emphasis on the intrinsic interactions between CQDs and biological targets. In addition, the challenges associated with the clinical translation of CQDs are highlighted, and new perspectives are provided from both macroscopic and microscopic views to guide the development of next‐generation CQDs and future clinical applications.

## 2 Introduction to Carbon Quantum Dots

Carbon quantum dots have shown unique advantages in biomedical applications due to their excellent physicochemical properties. Their functions are closely related to their surface chemical composition and internal structures. Different synthesis strategies and precursor conditions can produce CQDs with different sizes, surface functional groups, and structural features, resulting in CQDs with distinct functional properties. Therefore, before further exploring the biomedical applications and underlying mechanisms of CQDs, it is necessary to clarify their basic properties and functional characteristics and understand how different synthesis strategies affect their biological behaviors. In addition, differences in elemental composition give metal and nonmetal CQDs distinct functional properties and biosafety profiles. Establishing reasonable CQD selection principles based on these differences can help effectively link material properties with specific clinical applications and provide a theoretical basis for further investigation of their underlying mechanisms.

### 2.1 Characteristics and Functions of Carbon Quantum Dots

#### 2.1.1 Physicochemical Properties

CQDs are stable zero‐dimensional carbon‐based nanomaterials that typically consist of spherical carbon nanoparticles with sizes below 10 nm [13]. Their unique electron‐transfer properties and redox‐regulation capabilities enable them to participate in the generation or scavenging of reactive oxygen species (ROS), thereby influencing cellular signaling, immune regulation, and tissue microenvironment remodeling. These properties allow CQDs to provide regulatory effects during tissue regeneration [14, 15]. The carbon core of CQDs mainly contains sp^2^/sp^3^ hybridized structures. Their electronic structures and defect states provide tunable optical properties, allowing them to generate stable fluorescence signals. Therefore, CQDs have been widely used for bioimaging and dynamic monitoring of biological processes [16]. In addition, the surfaces of CQDs are enriched with active functional groups, including hydroxyl, carboxyl, and amino groups. These groups can be further modified through covalent or noncovalent interactions, enhancing the binding ability of CQDs with drugs, nucleic acids, and other biomolecules. This provides a favorable structural basis for precision delivery [17, 18]. Collectively, these characteristics make CQDs promising candidates for tissue regeneration and precision therapy.

In recent years, increasing evidence has shown that the physicochemical properties of CQDs can influence their biological behaviors. Specifically, smaller CQDs are more readily internalized by cells and can induce rapid and sustained biological effects [19]. The positive surface charge of CQDs enables electrostatic interactions with nucleic acids, thereby enhancing their gene delivery capability [20]. However, an increased density of positive charges strengthens electrostatic interactions with cell membranes, resulting in enhanced cellular uptake and further amplification of their biological functions [21, 22, 23]. In addition, surface modification of CQDs through the introduction of specific chemical groups can regulate their fluorescence properties and reduce potential cytotoxicity [19]. Beyond surface chemical properties, the internal structures and electronic characteristics of CQDs also play important roles in determining their biological functions. The conjugated structures formed by sp^2^ hybridized regions within CQDs enhance light absorption and promote photoinduced electron transfer. In contrast, an increased proportion of sp^3^ structures is positively associated with the number of surface functional groups, affecting the solubility and functionalization capacity of CQDs. However, an excessively high sp^3^ content may reduce their photocatalytic activity [24]. The sp^2^/sp^3^ hybridization ratio directly determines the photothermal conversion performance and ROS generation efficiency of CQDs [25]. Moreover, an increased band gap makes electron transitions more difficult, resulting in reduced ROS generation capability [23]. However, when CQDs possess similar particle sizes and surface charges, increased surface polarity may reduce their ability to cross biological barriers, thereby limiting their biomedical applications. Therefore, the close relationship between the microscopic physicochemical structures of CQDs and their biological properties provides an important theoretical basis for the rational design and precise application of CQDs.

#### 2.1.2 Biocompatibility and Biodegradability

CQDs possess excellent biocompatibility and biodegradability, which enable their application in the diagnosis and treatment of various diseases. Composed mainly of carbon, CQDs exhibit stable properties and a low tendency to generate toxic byproducts. Additionally, their extremely small size and high permeability reduce physical stimulation on cell membrane surfaces, preventing excessive activation of the immune system [26]. Notably, the surfaces of CQDs contain abundant functional groups, including amino, thiol, carboxyl, and hydroxyl groups. Hydrophilic groups improve the water solubility of CQDs, allowing them to disperse more effectively in biological fluids and reducing adverse effects caused by precipitation and aggregation [27].

The surface functional groups are also closely related to the degradation of CQDs. Enzymes and ROS within an organism can react with these functional groups and the carbon core [28]. Once degraded into small molecular substances, CQDs can be excreted from the body through respiratory, renal, and hepatic pathways. This degradation process helps prevent potential toxicity from excessive accumulation in the body, further enhancing their biosafety [29].

### 2.2 Synthesis of CQDs

#### 2.2.1 Top‐Down Approach

The top‐down method for synthesizing CQDs involves the decomposition of larger precursor materials into nanoparticles. Common top‐down techniques include chemical oxidation, arc discharge, laser ablation, and ultrasonic passivation [30]. The chemical oxidation method entails treating carbon materials with strong oxidizing acids to oxidatively decompose them into small CQDs. This method often produces CQDs with strong photoluminescence and a diverse range of functional groups [31]. The arc discharge method, which was used during the initial discovery of CQDs, can yield high‐quality CQDs. However, its relatively high cost makes it unsuitable for large‐scale production [32]. Laser ablation is simple and environmentally friendly, utilizing a high‐energy laser beam to irradiate carbon precursors, causing them to evaporate at elevated temperatures and subsequently form CQDs in the surrounding medium [33]. Ultrasonic passivation enables precise control over the size of CQDs, improving their uniformity and stability by adding a passivator and employing ultrasonic treatment [30]. Overall, however, the top‐down approach is less commonly employed in the synthesis of CQDs compared with the bottom‐up approach due to its low yield and stringent reaction conditions.

#### 2.2.2 The Bottom‐Up Approach

In contrast to the top‐down approach, the bottom‐up method of synthesizing CQDs involves the polymerization and transformation of small molecular precursors into CQDs under specific conditions, primarily including methods such as pyrolysis, hydrothermal/solvothermal methods, microwave methods, and template methods.

Pyrolysis involves heating carbon‐containing materials to induce thermal decomposition, followed by polymerization to produce CQDs; it is easy to operate and cost‐effective [31]. In the hydrothermal/solvothermal method, the precursor material is placed in water or a solvent for a high‐temperature and high‐pressure reaction, resulting in CQDs that typically exhibit better solubility [34]; it is simple and commonly used, making it an ideal technique for preparing CQDs. Microwave methods use microwave energy to quickly heat the precursor, facilitating its decomposition and subsequent generation of CQDs, though size control of the resulting CQDs remains a challenge [35]; nevertheless, microwave methods enable faster synthesis without the need for organic solvents. The template method uses specific support materials as templates to better control the size and shape of the CQDs. The template is then removed through high‐temperature treatment or strong acid and alkali processes [36]. This method can yield uniform CQDs; however, incomplete removal of the template may impact the purity and performance of the CQDs. Due to lower preparation costs, precise control over carbon dot size, and ease of surface functionalization, the bottom‐up approach is widely employed in the field of biomedicine.

### 2.3 Classification of CQDs

#### 2.3.1 Metal CQDs

The presence of multiple binding sites on the surface of CQDs allows for the introduction of metal elements through reactions between metal ions and surface functional groups, resulting in the formation of Metal CQDs. Metal ions, acting as electron donors, can regulate charge density and charge transfer after the introduction of CQDs, thereby enhancing their performance and photoluminescence efficiency, leading to the application of metal CQDs in biosensing, bioimaging, photodynamic therapy (PDT), and photothermal therapy (PTT) [37]. The introduction of metal elements can also endow CQDs with strong catalytic performance, allowing them to kill bacteria by promoting the production of ROS. Moreover, Metal CQDs can effectively activate signaling pathways related to tissue regeneration. In particular, the combination of CQDs with metals that possess osteogenic potential, such as Cu and Fe, further enhances their capability to promote bone regeneration [38]. However, it is important to note that the introduction of metal ions increases the overall volume of CQDs, which can complicate their metabolism. While current studies suggest that Metal CQDs are effectively cleared from the body via renal metabolic pathways, the dosage of metal doping and its potential long‐term toxicity require careful consideration.

#### 2.3.2 Nonmetal CQD

Nonmetal CQDs are composed of carbon along with nonmetal elements such as hydrogen, oxygen, nitrogen, and sulfur. They represent the fundamental form of CQDs and can be easily prepared from abundant and inexpensive raw materials. Additionally, the process of doping nonmetal elements into CQDs is relatively straightforward. Heteroatoms used for doping, such as nitrogen, sulfur, boron, and selenium, can modify the framework structure and surface functional groups of CQDs [39, 40]. The doped CQDs can be categorized into monoheteroatomic, diatomic co‐doped, and triatomic co‐doped CQDs based on the number of doping heteroatoms added [41].

Nitrogen doping can enhance the fluorescence efficiency of CQDs and improve their biocompatibility. Sulfur doping significantly enhances the chemical stability of CQDs and promotes their binding to cell surfaces [40]. Boron doping can improve the conductivity and catalytic activity of CQDs [42].

Compared with metal CQDs, nonmetal CQDs are easier to produce on a large scale. Most notably, they often demonstrate better biocompatibility and lower toxicity. In the context of tissue regeneration, nonmetal CQDs can be widely used to induce cell proliferation and differentiation and help regulate ROS and the immune microenvironment, presenting promising application prospects.

#### 2.3.3 Selection Principles of Different CQD Types for Biomedical Applications

Metal CQDs and nonmetal CQDs exhibit distinct biomedical functions due to differences in their elemental compositions and electronic structures. Metal CQDs generally possess tunable electronic structures and enhanced optical properties. The introduction of metal elements can regulate the electronic structure and surface active sites of CQDs, thereby improving light absorption, electron transfer, and catalytic activity [38]. However, the potential release of metal ions and their long‐term biosafety remain important concerns that require further evaluation. In contrast, nonmetal CQDs avoid the potential risks associated with metal ion release and generally exhibit better biocompatibility and lower potential toxicity. In addition, their functions can be optimized through the regulation of heteroatom doping, such as nitrogen, sulfur, and phosphorus, and surface functional group modification [43, 44].

For clinical translation, the selection of CQD types and structures should be more precisely matched according to specific biomedical requirements. For tissue repair processes that require long‐term biocompatibility, mild ROS regulation, and modulation of the tissue microenvironment, nonmetal CQDs generally show greater advantages. The introduction of heteroatoms such as nitrogen, sulfur, and selenium can regulate the electronic structure, enhance the *sp* ^2^‐conjugated framework of CQDs, and further promote electron delocalization. Together with appropriate surface defects, these modifications can regulate electron transfer efficiency and optimize redox activity [45]. These structural characteristics allow CQDs to maintain a dynamic balance between ROS generation and scavenging, thereby achieving mild and controllable ROS regulation. Moreover, surface functional groups enhance interactions between CQDs and cells, improving tissue regeneration efficiency. CQDs enriched with hydroxyl, carboxyl, and amino groups generally exhibit good hydrophilicity and biocompatibility and can serve as binding sites for proteins, growth factors, and other functional components [2]. For infected wounds or applications requiring enhanced catalytic therapy, iron‐ or magnesium‐doped CQDs can utilize the catalytic activity provided by metal elements to achieve synergistic antibacterial and tissue regeneration effects [2, 46]. In bioimaging applications, nonmetal CQDs with high quantum yields, excellent photostability, and near‐infrared emission properties are preferred [47]. However, for applications requiring magnetic resonance imaging or multimodal imaging, Metal CQDs containing magnetic elements or high atomic number elements may be more suitable [48]. In drug delivery systems, nonmetal CQDs with abundant hydroxyl, carboxyl, and amino groups can be selected for drug loading through electrostatic interactions or covalent coupling, thereby improving delivery efficiency and targeting ability [49]. For delivery systems requiring combined therapy or stimulus‐responsive release, metal ions can be further introduced to integrate catalytic functions with therapeutic effects [50]. In biosensing applications, the requirements for CQDs mainly focus on signal output capability, molecular recognition ability, and detection stability. Therefore, the selection of CQDs should not be determined solely by the presence or absence of metal elements, but should also consider their optical properties, electrochemical behaviors, and surface chemical characteristics. CQDs with high quantum yields, excellent photostability, abundant surface active sites, and tunable electronic structures can generally achieve more efficient signal responses and more accurate molecular detection, making them suitable for the construction of high‐performance biosensing platforms [51]. Precise selection of CQD types based on specific clinical application scenarios can help establish a better match between structural characteristics and functional requirements, thereby maximizing the potential of CQDs in tissue repair and precision therapy. However, the current selection of CQDs still largely relies on previous experience, and systematic principles for structural design and clinical application standards remain lacking. Future studies should integrate tissue microenvironment characteristics and clinical therapeutic goals to establish more detailed and rational application strategies. In addition, standardized evaluation systems and long‐term safety studies are required to promote the transition of CQDs from experimental research toward clinical applications.

## 3 Biomedical Applications of CQDs

CQDs have shown great potential in biomedical applications, particularly in tissue regeneration and precision therapy. Specifically, CQDs participate in tissue repair and regeneration by regulating cellular functions and the tissue microenvironment. Their applications in the regeneration of connective tissues, subcutaneous tissues, and solid connective tissues have attracted increasing attention. Meanwhile, the excellent optical properties, efficient transmembrane capability, and functionalizable surfaces of CQDs provide unique advantages in precision therapy, enabling high‐contrast bioimaging, precise delivery of therapeutic agents, and sensitive detection of biomolecules.

### 3.1 Applications of CQDs in Tissue Regeneration

CQDs have demonstrated broad application potential in promoting the regeneration of connective tissues, subcutaneous tissues, and solid connective tissues. Connective tissues can be classified into various types, including loose connective tissue, dense connective tissue, adipose tissue, blood, bone tissue, and cartilage tissue. Among them, bone and cartilage tissues are considered solid connective tissues. CQDs can directly promote tissue regeneration by regulating the expression of related genes and signaling pathways. In addition, they can indirectly enhance regeneration through antibacterial effects, immune regulation, and ROS scavenging. The following table systematically summarizes the recent applications of CQDs in different tissue regeneration processes, providing a reference for further research and application of CQDs in the field of tissue regeneration.

#### 3.1.1 Connective Tissue and Subcutaneous Tissue

Connective and subcutaneous tissues are essential for maintaining tissue structural integrity. Their repair and regeneration involve multiple processes, including fibroblast proliferation, migration, and differentiation, as well as angiogenesis, which together contribute to wound protection, tissue reconstruction, and restoration of tissue flexibility. Recent studies have shown that CQDs have considerable potential for repairing skin, wounds, and other soft tissue injuries. They can promote tissue regeneration through multiple mechanisms, including restoring cellular functions, regulating oxidative stress and inflammatory responses, and inhibiting bacterial infection. Table 1 summarizes representative studies of CQDs in connective and subcutaneous tissue regeneration in recent years and further categorizes them according to their precursors, types, synthesis methods, effects, and associated signaling pathways.

**TABLE 1:** Representative studies of CQDs in connective and subcutaneous tissue regeneration.

Connective tissue and subcutaneous tissue							
Precursor	Classification	Methods	Application	Effect	Pathways	Mechanism of action	Reference
Ascorbic acid	Nonmetal CQD	Hydrothermal/solvothermal	Dentin–pulp complex, blood vessels	Induces autophagy, ECM secretion, and angiogenesis	PI3K–AKT–MTOR signaling pathway	Direct	[52]
Citric acid, urea	Nonmetal CQD	Hydrothermal/solvothermal	Epidermal layer	Promotes adhesion, proliferation, and re‐epithelialization; antibacterial	—	Direct and indirect	[53]
Quercetin	Nonmetal CQD	Hydrothermal/solvothermal method	Nervous tissue	Promotes Schwann cell proliferation, migration, and differentiation; promotes nerve regeneration	NT‐3, BDNF, VEGFA, NGF	Direct	[54]
Carboxymethyl cellulose, citric acid, and urea	Nonmetal CQD	Hydrothermal/solvothermal method	Skin fibroblasts	Promotes fibroblast proliferation; antibacterial; promotes infected wound healing	TGFβ	Direct and indirect	[55]
Resveratrol	Nonmetal CQD	Hydrothermal/solvothermal method	Skin, blood vessels	Promotes angiogenesis; beneficial for wound healing	VEGF pathway	Direct	[56]
Curcumin	Nonmetal CQD	Hydrothermal/solvothermal and microwave hybrid methods	Skin	Induces angiogenesis and epithelial regeneration; promotes healing of *Staphylococcus aureus*/*Escherichia coli*‐infected wounds	—	Direct and indirect	[57]
*D*‐glucose and *L*‐aspartic acid	Nonmetal CQD	Pyrolysis	Skin	Anti‐*Staphylococcus aureus*, promotes infected skin wound healing	—	Indirect	[58]
Goji berry oligosaccharides	Nonmetal CQD	Hydrothermal/solvothermal method	Nerve	Induces differentiation of neural stem cells into mature neurons	*Neurog2, Tubb3, Gap43, Syn1, Snap25, Nefh, Gad1, Grin1*, and *Sox*	Direct	[59]
Oxalic acid, nitrogen compound	Nonmetal CQD	Hydrothermal/solvothermal method	Skin	Anti‐*Staphylococcus aureus* and promotes burn‐wound healing	—	Indirect	[60]
Chitosan, ferrous sulfate, heptahydrate, p‐phenylenediamine	Metal CQDs	Hydrothermal/solvothermal method	Skin	Anti‐*Staphylococcus aureus*, induces hair follicle and blood vessel formation, promotes wound healing	—	Direct and indirect	[61]
Glutathione, polyethylene glycol	Nonmetal CQD	Hydrothermal/solvothermal method	Tendon	Anti‐inflammatory, antioxidant, promotes tendon healing, and reduces tendon adhesions	NRF2–HO‐1 signaling pathway	Indirect	[62]
Citric acid, guanidine hydrochloride, copper chloride	Metal CQDs	Hydrothermal/solvothermal method	Diabetic skin	Scavenges O2·− and ·OH; reduces ROS; antioxidative stress; promotes wound healing	—	Indirect	[63]

The regenerative effects of CQDs on connective and subcutaneous tissues involve multiple levels of regulation. CQDs can directly act on fibroblasts and endothelial cells by regulating multiple signaling pathways, while also improving the local microenvironment through antibacterial activity, modulation of inflammatory responses, and removal of excess ROS. These effects together create favorable conditions for the restoration of cellular functions and tissue regeneration. Notably, persistent infection and prolonged inflammation in chronic wounds make tissue repair and regeneration particularly challenging. Recent studies have further shown that CQDs can be integrated with smart responsive designs to achieve precise and dynamic regulation of the wound microenvironment, providing new strategies for the targeted treatment and tissue regeneration of chronic wounds [64].

#### 3.1.2 Solid Connective Tissue

Solid connective tissues, such as bone and cartilage, primarily provide mechanical support and maintain structural stability in the body. Their regeneration depends on cell proliferation and differentiation and is also influenced by extracellular matrix formation, inflammatory responses, and the local tissue microenvironment. CQDs can regulate the biological behavior of mesenchymal stem cells to promote osteogenic or chondrogenic differentiation, while indirectly creating favorable conditions for the regeneration of solid connective tissues. Table 2 summarizes representative studies of CQDs in the regeneration of solid connective tissues, including bone and cartilage, in recent years, and summarizes their material characteristics, effects, and molecular mechanisms.

**TABLE 2:** Representative studies of CQDs in solid connective tissue regeneration.

Solid connective tissue							
Precursor	Classification	methods	Application	Effect	Pathways	Pleiotropic effects	Reference
Clove residue	Nonmetal CQD	Hydrothermal/solvothermal method	Human bone marrow‐derived mesenchymal stem cells	Osteogenic differentiation	WNT/β‐catenin, ERK, and BMP signaling pathway; *Gsk3b*	Direct and indirect	[65]
Garlic peel	Nonmetal CQD	Hydrothermal/solvothermal method	Cartilage	Chondrocyte differentiation and cartilage regeneration	COLI	Direct	[66]
Ascorbic acid, polyethylenimine	Nonmetal CQD	Microwave assisted	Skull	Promotes osteoblast proliferation, differentiation, and mineralization; promotes bone defect repair and regeneration	PERK–eIF2α–ATF4 signaling pathway; *Runx2*, *Alpl*	Direct	[67]
Arginine	Nonmetal CQD	Pyrolysis	Thighbone	Antibacterial; induces bone regeneration	*Il10*	Direct and indirect	[68]
Ascorbic acid	Nonmetal CQD	Microwave method	Tibia, skull	Delivers miR‐2861; promotes osteogenic differentiation in vitro and new bone regeneration in vivo	—	Indirect	[69]
Anhydrous citric acid	Nonmetal CQD	Pyrolysis	Thighbone	Promotes M1 to M2 macrophage polarization; lethal to drug‐resistant S. aureus; promotes osteoblast mineralization and new bone formation	OCN, Runx2, ALP, OPG, *Il4, Il10*, and *Col1a1*; TNF, *Il6*, and *Il1β*	Direct	[70]
Chitosan, acrylamide, ammonium persulfate	Nonmetal CQD	Hydrothermal/solvothermal method	Cartilage of the knee joint	Loaded with mulberry xanthin; protects cartilage; promotes cartilage regeneration	—	Indirect	[71]
Citric acid	Nonmetal CQD	Pyrolysis	Stem cell osteogenic differentiation	Promotes osteogenic differentiation of stem cells from human exfoliated deciduous teeth; improves calcification	*Ocn, Runx2, Col1a1*, and WNT‐β‐catenin	Direct	[72]

CQDs show considerable potential for the regeneration of solid connective tissues, including bone, cartilage, and periodontal tissues, by coordinating regenerative processes through both direct and indirect effects. However, the biological effects of CQDs are strongly dependent on their material properties. Future studies should further clarify how CQD precursors and structural characteristics are related to tissue‐specific biological effects and optimize CQD design according to the type of tissue and its regenerative requirements.

### 3.2 Applications of CQDs in Precision Therapy

CQDs possess excellent fluorescence properties, abundant surface functional groups, good biocompatibility, and facile functionalization capabilities. These characteristics enable CQDs to serve not only as bioimaging probes for precise disease localization but also as nanocarriers to improve drug delivery efficiency and further construct highly sensitive biosensing platforms for dynamic monitoring of disease‐related molecules. Therefore, CQDs have gradually evolved from single diagnostic or therapeutic materials into integrated precision therapy platforms combining diagnosis, treatment, and monitoring, providing new technological approaches for the development of precision medicine.

#### 3.2.1 Bioimaging

CQD‐based bioimaging technology relies on optical contrast signals and enables high‐resolution observation at the molecular, cellular, and living organism levels. It has important applications in early disease detection and image‐guided therapy [73]. The imaging performance of CQDs mainly originates from their unique electronic structures. The fluorescence emission of CQDs is primarily derived from the sp^2^ conjugated structures within the carbon core, which facilitate electron delocalization and stable photoinduced electron transitions. In addition, surface defect states and multiple surface emission states induced by oxygen‐ and nitrogen‐containing functional groups further regulate their fluorescence behaviors [73, 74].

Currently, the applications of CQDs in bioimaging have gradually expanded from simple cellular fluorescence labeling to in vivo disease diagnosis. At the cellular level, CQDs can enter cells and achieve organelle‐specific localization. Studies have shown that CQDs with highly similar surface chemical properties can achieve highly specific imaging of mitochondria and lysosomes by regulating their polarity. This process does not alter organelle membrane potential, making CQDs suitable for long‐term dynamic tracking and physiological mechanism studies, and providing new targets for organelle labeling [75]. By further introducing targeting ligands, CQDs can selectively recognize tumor cells or inflammatory regions, enabling precise imaging of disease sites. For example, AS1411‐Gd‐CQDs can selectively accumulate in tumor regions by targeting nucleolin, which is highly expressed in tumor cells, via the AS1411 aptamer. This enables dual‐modal fluorescence and magnetic resonance imaging for precise tumor visualization [76]. During inflammation, large amounts of positively charged proteins, such as transferrin, accumulate at inflammatory sites. Negatively charged CQDs can be attracted to these regions through electrostatic interactions and achieve in vivo imaging of colitis lesions based on their stable red fluorescence signals [77]. However, conventional visible‐light‐emitting CQDs are still limited by insufficient tissue penetration depth and background fluorescence interference. In recent years, researchers have developed near‐infrared‐emitting CQDs through heteroatom doping, such as nitrogen and sulfur, and structural regulation [78]. With the development of near‐infrared emission and multiphoton imaging technologies, CQDs have gradually been applied to imaging of living organs and animal models. Phenylene diamine‐derived CQDs loaded into vaterite composites achieved real‐time in vivo two‐photon tracking of mouse cerebral blood vessels due to the two‐photon absorption capability of CQDs in the near‐infrared region, reaching approximately 50 GM. This provides a feasible imaging tool for investigating cerebrovascular‐related diseases and blood‐brain barrier permeability [79].

Although CQDs have gradually developed from traditional cellular fluorescent labeling materials into nanoimaging tools with disease localization and diagnostic potential, their further clinical translation still faces several challenges. Most current CQDs rely on visible light or first near‐infrared (NIR‐I) emission, which is susceptible to light scattering and autofluorescence interference in complex tissues, resulting in limited imaging depth. Moreover, current CQD‐based imaging mainly evaluates fluorescence intensity changes as the primary indicator, while the quantitative relationship between signal variation and CQD concentration, tissue distribution, and biological processes remains insufficiently investigated, limiting their transition toward precise measurement. Future studies should focus on regulating CQD structures, heteroatom doping, and surface electronic environments to develop new CQDs with second near‐infrared (NIR‐II) emission and enhanced tissue penetration ability, thereby improving imaging resolution under complex physiological conditions. In addition, the integration of artificial intelligence‐based analysis, multimodal imaging, and standardized quantitative evaluation systems may facilitate the transition from image acquisition to precise assessment of disease states.

#### 3.2.2 Drug Delivery

Traditional drug therapy is often limited by poor drug solubility, short circulation half‐life, and uneven tissue distribution. These limitations make it difficult to achieve effective accumulation and spatially and temporally controlled drug release in complex diseases, resulting in reduced therapeutic efficacy and increased damage to normal tissues [80]. Therefore, the development of novel drug delivery systems with targeted delivery capabilities is essential.

The surfaces of CQDs contain abundant active sites, including carboxyl, hydroxyl, and amino groups, which enable the loading of therapeutic molecules through electrostatic interactions, covalent coupling, physical encapsulation, or conjugation strategies [81]. Meanwhile, the small size of CQDs facilitates cellular uptake, allowing them to serve as important carriers for drug transport and intracellular delivery [82]. Taking advantage of their easily modifiable surfaces, researchers can further introduce targeting ligands or functional components to enable CQDs to actively recognize specific cells or disease sites. For example, folate receptors and heparan sulfate proteoglycan receptors, which are highly expressed in tumor cells, can serve as targets for Ex@MTX‐CQD. Through the synergistic effects of folic acid and exosomes, Ex@MTX‐CQD achieves methotrexate loading and enhances its specific delivery and uptake in tumor cells, thereby improving antitumor therapeutic effects [83]. Based on their targeting ability, CQDs have been further applied for the delivery of various therapeutic molecules, including nucleic acids and proteins, in addition to conventional small‐molecule drugs. Because nucleic acids are easily degraded and have limited cellular uptake ability, stable and efficient delivery systems are required [84]. CQDs can achieve stable binding with nucleic acids through surface modification and promote intracellular release after endocytosis, making them promising materials for gene regulation therapy. Specifically, positively charged amino‐functionalized CQDs can form complexes with nucleic acids through electrostatic interactions. After modification with the AS1411 aptamer, these CQDs enable targeted delivery to hepatocellular carcinoma cells, resulting in efficient intracellular uptake of siRNA and silencing of the target gene FMR1 [85]. Unlike nucleic acids, which mainly regulate cellular functions indirectly through gene expression, proteins can directly participate in cellular signaling processes. However, their large molecular size, strong hydrophilicity, and limited ability to cross cell membranes, together with immune clearance barriers, often require delivery systems to achieve safe and effective intracellular transport [86]. Relying on abundant surface active sites, CQDs can load proteins while maintaining their biological activity, providing a new carrier platform for precision protein therapy. MCQDs can bind with IGF‐1 proteins through noncovalent supramolecular interactions, including electrostatic interactions and hydrogen bonding. This interaction protects IGF‐1 from enzymatic degradation and enables intracellular protein delivery as well as hydrogel‐mediated sequential release, thereby effectively promoting anti‐inflammatory and osteogenic effects [87]. In addition, CQDs possess intrinsic optical properties that allow them to participate in multifunctional therapy. By utilizing the photothermal conversion capability of CQDs, local temperature elevation can be induced to achieve photothermal therapy. When combined with targeted chemotherapeutic drug delivery, this strategy can further improve antitumor therapeutic efficacy [88].

After achieving disease localization and therapeutic molecule delivery, further controlling drug release at the target site is a key factor for improving the precision of CQD‐based delivery systems. Compared with traditional delivery methods that mainly rely on passive drug diffusion, responsive CQD systems can utilize disease‐associated microenvironmental features to trigger drug release, transforming drug delivery into a spatially and temporally selective regulatory process. Currently, most CQD‐based delivery systems still rely on a single external stimulus, whereas real disease microenvironments usually involve coordinated changes among multiple factors and dynamic regulation. Future responsive CQD delivery systems should move from single‐stimulus responses toward multifactor synergistic responses and intelligent release regulation. By integrating the surface chemical structures, electronic properties of CQDs, and disease microenvironmental characteristics, predictable release behaviors can be established to achieve precise therapeutic molecule delivery and dynamic regulation of treatment outcomes.

#### 3.2.3 Biosensing

During disease diagnosis and treatment, early and high‐resolution capture and quantification of disease biomarkers are essential for early warning and real‐time evaluation of therapeutic responses [89]. Compared with traditional monitoring methods, CQD‐based biosensing enables more sensitive, rapid, dynamic, and noninvasive monitoring of complex disease changes. In addition, CQDs exhibit better photostability and stronger resistance to photobleaching than conventional fluorescent dyes [90]. When target molecules interact with the surfaces of CQDs, detectable signals can be generated through mechanisms including fluorescence enhancement, fluorescence quenching, and electron transfer [91]. By regulating surface defects, heteroatom doping, and functional group composition, the sensitivity, selectivity, and response speed of CQDs can be further optimized, enabling molecular detection in complex biological environments [92].

Currently, CQD‐based biosensing has been applied to the detection of various disease‐related biomarkers. In metabolic diseases, CQDs can detect glucose, uric acid, and other metabolites through fluorescence or electrochemical signal changes, providing new approaches for early disease screening. Because boronic acid groups can interact with cis‐diol structures in glucose molecules to form stable cyclic complexes, introducing boronic acid groups onto CQD surfaces can enhance fluorescence emission and enable quantitative glucose monitoring [93]. In diseases associated with redox imbalance, CQDs can respond to changes in glutathione (GSH) levels within the microenvironment, thereby reflecting tissue oxidative status and inflammation levels. In the presence of GSH, thiol groups can undergo addition reactions with unsaturated double bonds on the surface of DB‐CQDs. This process inhibits electron transfer between surface amino groups and the carbon core, resulting in enhanced fluorescence signals and enabling rapid and sensitive detection of GSH fluctuations in living cells [94]. CQD‐based sensing technologies can also be further applied to detect disease biomarkers such as nucleic acids and proteins, providing new strategies for early disease screening and classification. Due to their excellent fluorescence properties and modifiable surface structures, tremella‐derived CQDs can serve as fluorescent signal output elements. In the presence of target nucleic acids, these CQDs undergo dynamic fluorescence quenching, enabling quantitative detection of specific miRNAs in hepatocellular carcinoma cell lines and providing potential applications for early cancer diagnosis and classification [95]. For protein biomarker detection, CQD‐modified sensing complexes can achieve highly sensitive detection of lung cancer‐related biomarkers CYFRA 21‐1 and NSE antigens in serum, while exhibiting good anti‐interference capability and stability [96]. With the development of liquid biopsy technologies, exosomes have become important diagnostic biomarkers due to their abundant disease‐related information [97]. However, the application of CQDs in exosome detection remains at an exploratory stage. Developing highly specific exosome sensing systems will become an important research direction for future precision diagnosis. Beyond ex vivo biomarker analysis based on body fluid samples, CQD‐based sensing technologies have also shown increasing potential for dynamic in vivo monitoring. Fe‐CQD probes can achieve noninvasive, real‐time, and precise monitoring of gastric pH values in mice, accurately reflecting physiological pH fluctuations during digestion [98].

With the development of artificial intelligence, flexible wearable devices, and miniaturized detection technologies, CQD‐based sensing has further advanced toward continuous monitoring and intelligent feedback. These systems enable long‐term real‐time monitoring of physiological indicators and disease‐associated microenvironments, providing data support for chronic disease management and personalized therapy. However, CQD‐based sensing technologies still face challenges, including insufficient long‐term stability in vivo and reduced signal accuracy under complex biological conditions. By integrating biosensing with drug delivery and responsive release systems, CQDs can further facilitate the construction of intelligent theranostic platforms with sensing, analysis, and regulation capabilities, enabling dynamic adjustment of therapeutic strategies according to disease status.

In recent years, CQDs have been widely applied in bioimaging, drug delivery, and biosensing due to their unique optical properties, tunable surface structures, and excellent biocompatibility. These advantages provide important support for precision diagnosis and therapy, demonstrating broad biomedical application prospects. However, compared with these applications, CQDs have been investigated more extensively in tissue regeneration and exhibit greater potential for clinical translation. Therefore, the following sections will systematically discuss the direct mechanisms and indirect regulatory mechanisms underlying CQD‐mediated tissue regeneration.

## 4 Mechanisms of CQDs in Promoting Tissue Regeneration

Tissue regeneration involves not only cell proliferation, differentiation, and functional recovery but is also affected by persistent inflammation, oxidative stress, and microbial infection, which can impede the regenerative process. Therefore, promoting tissue regeneration requires not only activation of the intrinsic repair potential of regenerative cells but also remodeling of the local microenvironment to support tissue recovery. Benefiting from their unique physicochemical properties, CQDs can promote tissue regeneration through the coordinated activation of cellular functions and remodeling of the local microenvironment. The following sections systematically describe the mechanisms by which CQDs promote tissue regeneration through direct and indirect actions.

### 4.1 CQDs Directly Promote Tissue Regeneration

At the cellular level, CQDs can regulate intracellular signaling networks and cellular functions to activate the proliferation, migration, and directed differentiation of key cells involved in tissue regeneration, thereby promoting tissue reconstruction. Fibroblast activation, angiogenesis, and mesenchymal stem cell differentiation are central processes in tissue repair and represent important mechanisms through which CQDs directly promote regeneration (Figure 1A–D).

**FIGURE 1:**
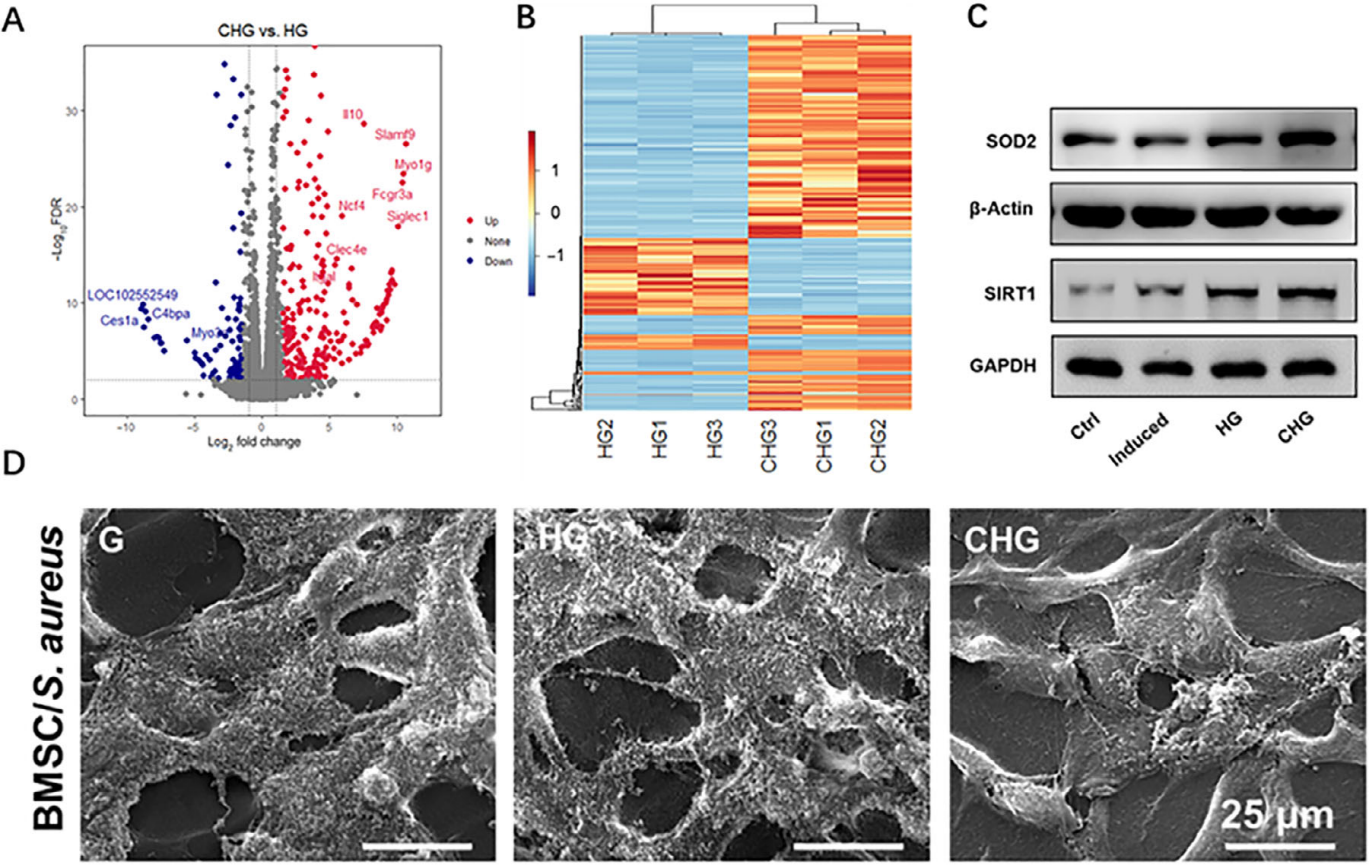
**Representative mechanistic evidence for CQDs in regulating cellular functions and the regenerative microenvironment. (**A, B) Transcriptomic analysis reveals CQD‐induced remodeling of cellular gene expression. (C) Changes in the expression of antioxidant‐related proteins in BMSCs following CQD treatment. (D) Morphological observation of the interaction between BMSCs and *Staphylococcus aureus* after CQDs treatment. Copyright Permission from Ref. [68].

#### 4.1.1 Fibroblast

Fibroblasts play a crucial role in tissue regeneration by synthesizing collagen fibers and forming the extracellular matrix, which aids in the formation of granulation tissue. They contribute to tissue reconstruction through three physiological processes: proliferation, migration, and differentiation (Figure 2).

**FIGURE 2:**
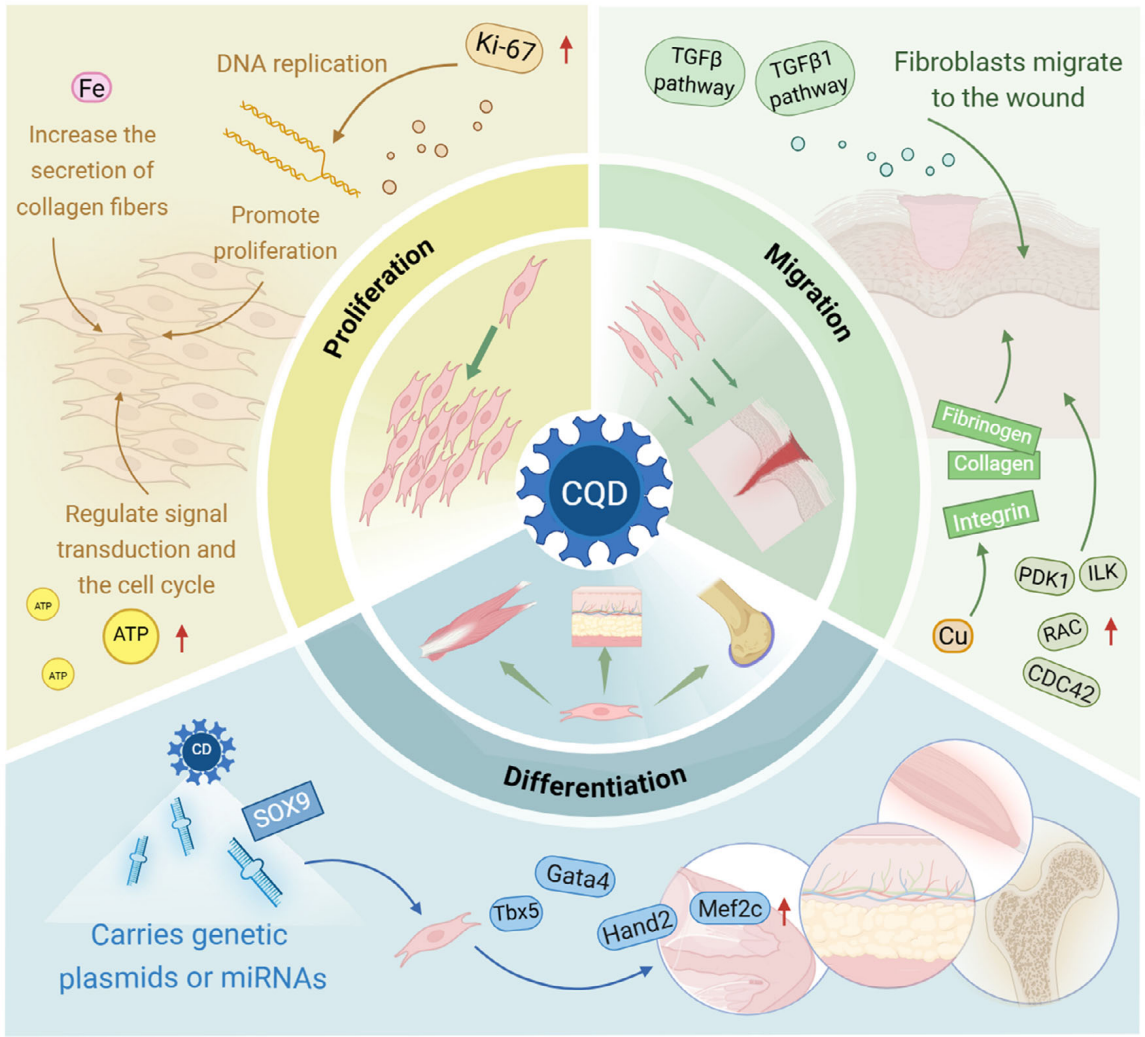
**Multifaceted regulation of fibroblast functions by CQDs through multiple signaling pathways**. Schematic illustration of CQD‐mediated regulation of fibroblast proliferation, migration, and differentiation, highlighting the key signaling pathways involved in these processes.

Proliferation: Fibroblasts provide the cellular foundation necessary for tissue regeneration. KI‐67, a nuclear protein, is essential in fibroblast proliferation and DNA replication. The synergistic effect of AgMOFN‐CQD and near‐infrared light can enhance KI‐67 expression and promote fibroblast proliferation [99, 100]. Additionally, ATP supplies energy to cells and regulates signal transduction and the cell cycle. AuAg‐CQD has been shown to increase ATP levels in fibroblasts, stimulating cell proliferation [101]. Furthermore, Fe‐CQD promotes fibroblast proliferation primarily due to the presence of iron, an important trace element that can easily enter fibroblasts, thereby inducing proliferation and significantly increasing collagen fiber secretion [102].

Migration: The migration of fibroblasts is essential for tissue regeneration, particularly in wound healing. This migration increases the number of surrounding cells, facilitating fibroblast involvement in the repair process. TGFβ1 can induce cell migration through specific receptors, and CQDs can stimulate fibroblast migration by activating the TGFβ pathway, accelerating wound healing [103]. Cu‐CQD also exhibits a strong ability to promote fibroblast migration, attributed to the enhancement of integrin, collagen, and fibrinogen expression by Cu, thereby facilitating tissue re‐epithelialization [104, 105]. TMP‐CQD can promote fibroblast migration by enhancing the expression of key genes, such as *Cdc42, Rac1, Pdk1*, and *Ilk*, which regulate cell migration [106].

Differentiation: Fibroblasts can differentiate into mature cells with specific functions, such as myofibroblasts, adipocytes, osteocytes, and chondrocytes. This differentiation is beneficial for restoring and enhancing the structure and function of regenerated tissue. Currently, CQDs are primarily used as delivery carriers; for instance, CQDs carrying the gene plasmid SOX9 can efficiently enter the cell nucleus and promote the differentiation of fibroblasts into chondrocytes [107]. Additionally, BP‐NCQD carrying miRNA can directly induce cardiac fibroblasts to transform into mature cardiomyocytes, significantly increasing the expression of *Gata4, Hand2, Mef2c*, and *Tbx5* [108]. Given that fibroblast differentiation provides specific cell types crucial for tissue repair and regeneration, future research should focus on the direct effects of CQDs on fibroblasts. Furthermore, the role of fibroblasts in immune regulation has gradually attracted attention, but the application of CQDs in this field is currently limited. Future research can specifically investigate the impact of CQDs on the immune regulatory functions of fibroblasts.

#### 4.1.2 Angiogenesis

The initial stages of angiogenesis involve the degradation of the basement membrane, during which tip cells differentiate in response to signaling factors, prompting adjacent cells to adopt a stalk cell phenotype. Subsequently, through the proliferation and migration of endothelial cells, new microvascular lumens gradually form, accompanied by the stabilization and maturation of blood vessels, ultimately establishing a functional vascular network [109, 110, 111]. Due to their exceptionally high permeability, CQDs can easily reach areas of vascular injury and influence angiogenesis by regulating the expression of related genes, thereby affecting the signaling pathways associated with blood vessel formation (Figure 3). The specific mechanisms are explained in detail below:

**FIGURE 3:**
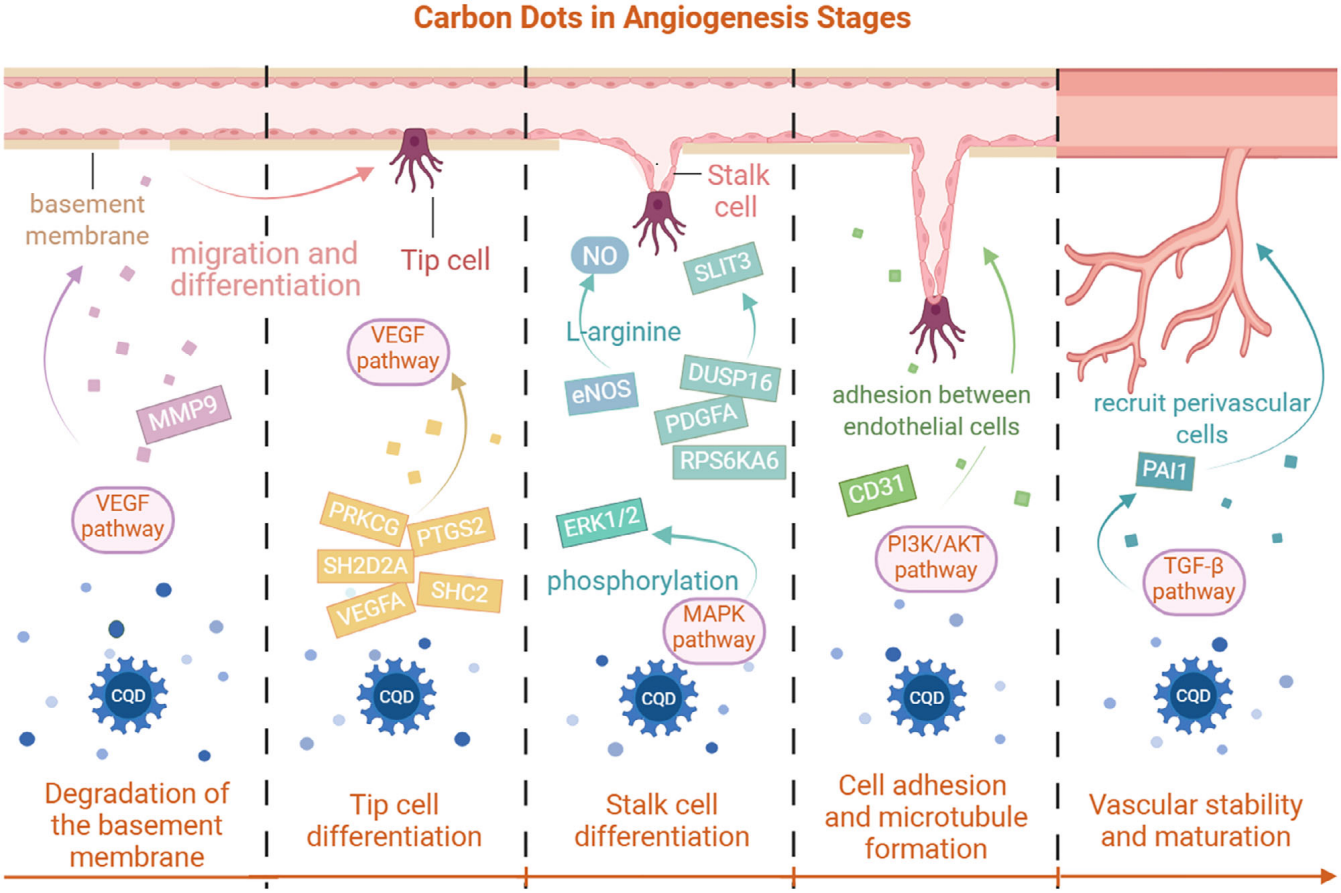
**CQDs exert multifaceted regulatory effects on vascular regeneration via dynamic modulation**. Schematic illustration of the effects of CQDs on angiogenesis‐related cells and signaling molecules at key stages of vascular regeneration.

Differentiation of tip cells: Vascular endothelial growth factor (VEGF), one of the most important angiogenic factors, stimulates endothelial cells to transform into tip cells, promoting the formation of new blood vessels. Research indicates that CQDs can significantly enhance VEGF expression, along with a marked increase in matrix metalloproteinase 9 (MMP9) expression, which can facilitate the degradation of the basement membrane and assist in the migration and differentiation of tip cells [112, 113]. BC/Cu(II)‐RCQD activates the VEGF pathway by upregulating genes such as *Vegfa, Prkcg, Jmjd7‐Pla2g4b, Shc2, Ptgs2, Sh2d2a*, and *Res‐CD*, while *Eucommia*‐derived CQDs have also been shown to promote VEGF expression [56, 114, 115].

Stalk cell proliferation: Endothelial nitric oxide synthase (eNOS) utilizes L‐arginine as a substrate to generate nitric oxide (NO), which promotes endothelial cell proliferation for blood vessel formation and regulates the expression of other angiogenesis factors [116]. Studies have demonstrated that GQDs‐BC can upregulate eNOS expression and promote cell proliferation [113]. Additionally, CQDs can activate MAPK pathways, promote ERK1/2 phosphorylation, and increase the expression of *Slit3* by upregulating *Pdgfa*, *Rps6ka6*, and *Dusp16*, thereby inducing stalk cell proliferation and promoting vascular formation [115, 117, 118].

Cell adhesion and microvascular lumen formation: Platelet endothelial cell adhesion molecule 1 (PECAM1/CD31) is critical in the angiogenesis process, promoting endothelial cell junction formation, migration, and adhesion, which are essential for the development of microvascular lumens [61]. Research indicates that H‐Zn/CQD, RES‐CQD, CQD‐Cu‐N, and EUO‐NAC‐CQD can enhance CD31 expression and promote adhesion between endothelial cells [56, 111, 114, 119, 120].

Stability and maturity: Previous studies have shown that sufficient expression of TGFβ can promote the secretion of plasminogen activator inhibitor 1 (PAI1), recruit perivascular cells, and is crucial for the stability and maturation of blood vessels [109]. Results indicate that CQD‐Cu‐N can promote TGFβ expression, while carbon quantum dots‐nitric oxide (CQD‐NO) can increase TGFβ levels in vascular endothelial cells by releasing NO, enhancing angiogenesis and matrix deposition, and contributing to the stable maturation of blood vessels [121, 122].

However, due to their small size, CQDs can easily pass through the dense vascular endothelium. Consequently, when they act on the tips of pre‐existing blood vessels to promote vascular regeneration, they may lack sufficient retention, leading to a deficiency in maintaining local drug concentrations. Future surface modifications to CQDs, such as encapsulating them with bioengineering membranes to enhance their retention at targeted sites or introducing tissue‐specific targeting molecules, such as mannose or folic acid, during the selection of carbon sources for carbon dot preparation, could strengthen their targeting effects on cells or tissues. These modifications aim to increase local drug concentrations and more effectively promote angiogenesis.

#### 4.1.3 Promotion of Mesenchymal Stem Cell Differentiation

Mesenchymal stem cells possess multidirectional differentiation potential, allowing them to transform into specific types of mature cells, thereby providing a cellular foundation for tissue regeneration. CQDs can promote the differentiation of mesenchymal stem cells by activating intracellular signaling pathways or regulating the selective expression of genes, thereby contributing to tissue regeneration (Figure 4).

**FIGURE 4:**
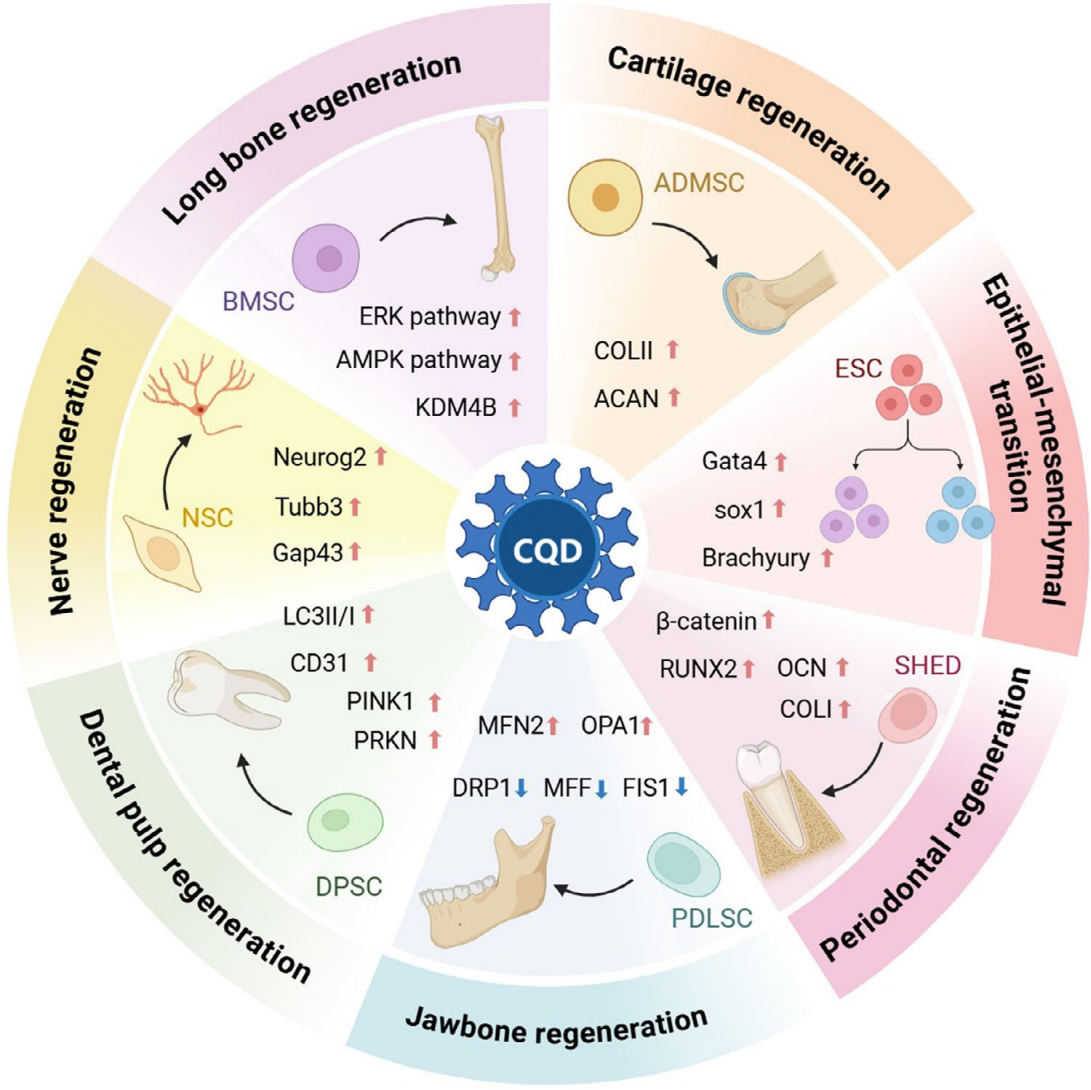
**Promotion of mesenchymal stem cell differentiation by CQDs**. Schematic illustration of the effects of CQDs on the expression of differentiation‐related genes and the subsequent differentiation of different types of mesenchymal stem cells.

##### 4.1.3.1 Bone Marrow Mesenchymal Stem Cells

In the context of bone injury models, the differentiation of bone marrow mesenchymal stem cells (BMSCs) plays a crucial role [123]. Research has shown that CQDs stimulate the differentiation of BMSCs into osteoblasts by increasing the expression of *Kdm4b* and regulating the expression of osteogenesis‐related factors, including *Runx2*, *Alpl*, *Opg*, *Opn*, and *Col1a1* [124, 125]. Furthermore, metformin CQDs can activate the ERK signaling pathway and its downstream target, the AMPK pathway, jointly regulating BMSC differentiation and metabolism [126]. CQDs also enhance the production of organic matter and the mineralization of BMSCs during their differentiation into osteoblasts. However, sulfonated glycosaminoglycan bioCQDs require the assistance of osteogenic induction medium to effectively promote osteogenic differentiation [127].

##### 4.1.3.2 Dental Pulp Stem Cells

Dental pulp stem cells (DPSCs), stem cells from human exfoliated deciduous teeth (SHED), and periodontal ligament cells (PDLCs) hold great potential for promoting the regeneration and repair of oral tissues. Both DPSCs and SHED derive from dental pulp tissue, share a common origin related to the mesenchymal cells of the dental papilla, and possess multi‐directional differentiation potential that facilitates the repair and regeneration of pulp and periodontal tissues. PDLCs originate from the periodontal ligament and arise from the mesoderm of the dental germ, playing a key role in the repair and regeneration of periodontal tissues. Experimental studies have demonstrated that CQDs can increase the expression of P62 and LC3II, thereby promoting autophagy and significantly enhancing the expression of odontogenic genes *Dspp*, *Dmp1*, *Sp7*, and *Runx2*, thus facilitating the differentiation of DPSCs into odontoblasts [128]. Additionally, other studies have shown that CQDs can promote the differentiation of DPSCs by increasing the expression of *Egf*, *Pecam1*, and *Col1a1* [129]. CQDs also regulate DPSC differentiation in vivo, with the specific mechanism related to the promotion of mitophagy; finally, CQDs can increase the expression levels of PINK1, PRKN, and P62, while elevating the LC3 II/I ratio [130].

##### 4.1.3.3 SHED

For SHED, in vitro results demonstrated that CQDs could upregulate the expression of *Ocn*, *Runx2*, *Col1a1*, and components of the WNT–β‐catenin signaling pathway, including *Ctnnb1*. This upregulation promotes the osteogenic differentiation of SHED and enhances calcification levels [72]. For PDLCs, CQDs combined with gold nanoparticles exhibited a synergistic effect, significantly enhancing the expression levels of *Osx* and *Runx2*, thus promoting the osteogenic differentiation of PDLCs [131]. Research indicates that Y‐GOQDs can regulate mitochondrial dynamics, enhancing the expression of fusion‐related proteins MFN2 and OPA1, inhibiting the expression of fission‐related proteins DRP1, MFF, and FIS1, and increasing the expression levels of *Osx*, *Runx2*, *Opn*, and *Ocn* to promote osteogenic differentiation of PDLCs [132].

##### 4.1.3.4 Adipose Tissue‐Derived Mesenchymal Stem Cells

Adipose tissue‐derived mesenchymal stem cells (ADMSCs) can differentiate into cartilage, playing a crucial role in the repair and regeneration of cartilage injuries. Peptide‐conjugated CQDs can induce adipose tissue‐derived mesenchymal stem cells to express cartilage‐related genes *Col2a1* and *Acan*, thereby promoting their differentiation into cartilage [133].

Nerve injuries severely impact bodily functions such as movement and sensation. Neural stem cells can differentiate into mature neurons when induced, thereby aiding in the repair and regeneration of nerve damage. Studies have shown that CQDs can increase the expression of *Neurog2*, *Tubb3*, *Gap43*, *Syn1*, *Snap25*, *Nefh*, *Gad1*, *Grin1*, *Sox*, *Dcx*, and induce the differentiation of neural stem cells into mature neurons [59].

In the context of tissue repair and regeneration, pluripotent stem cells, such as embryonic stem cells, can differentiate into all cell types within the body under specific conditions, which is crucial for the repair of various organs. Research findings indicated that 80 µg/mL NCQD could promote the expression of *Gata4*, *Sox1*, and *Brachyury*, inducing embryonic stem cell differentiation into endoderm and mesoderm cells [134].

However, the activation of different signaling pathways by CQDs exhibits variability. Future research should focus on identifying specific molecular targets and adjusting the distribution and quantity of functional groups on the surface of CQDs to achieve targeted modulation. This targeted approach will provide a theoretical basis for designing novel CQDs that facilitate the differentiation of induced stem cells and enhance the clinical applications of CQDs in tissue regeneration and repair.

### 4.2 CQDs Indirectly Promote Tissue Regeneration

Tissue regeneration does not rely on the isolated actions of a single cell; instead, it entails a complex biological process in which various functional cells interact and collaborate with microorganisms within their ecological niche. Concurrently, signaling factors, such as ROS, play a critical role in tissue regeneration.

ROS are a class of molecules or atoms formed by the incomplete reduction of oxygen and participate in various reactions both inside and outside the cell. The primary forms of ROS include H_2_O_2_, ·OH, ^1^O_2_, O2•−, 2,2’‐azinobis(3‐ethylbenzothiazoline‐6‐sulfonic acid) radical, and 1,1‐diphenyl‐2‐picrylhydrazyl radical (DPPH·), among others [135].

Intracellular ROS primarily originate from the mitochondria. During cellular respiration, the electron transport chain in the inner mitochondrial membrane generates a considerable amount of ROS. Additionally, NADPH oxidase is a crucial enzyme complex responsible for ROS production within cells [136]. Other cellular components, including the endoplasmic reticulum and various enzymes, can also produce ROS, which can diffuse across the cell membrane and influence the extracellular environment [137, 138].

#### 4.2.1 Bactericidal and Antibiofilm Mechanisms

A major challenge in the process of tissue regeneration is combating bacterial infections. Various types of bacteria and their biofilms can produce virulence factors that hinder wound healing. Common pathogenic bacteria, including both Gram‐positive and Gram‐negative strains, can exacerbate inflammatory responses, leading to impaired morphological and functional regeneration of tissues, and may even result in tissue necrosis and systemic infections [139]. In this context, CQDs exhibit strong lethal capabilities against most Gram‐positive bacteria and some Gram‐negative bacteria [140].

Biofilms serve as a breeding ground for bacteria, providing a protective barrier that resists adverse environmental conditions while promoting adhesion and tissue invasion, ultimately enhancing their pathogenicity [141, 142]. Due to their small size, CQDs possess exceptional tissue penetration capabilities, allowing them to overcome barriers associated with biological membranes to exert antimicrobial effects. In addition, the surface functional groups of CQDs demonstrate a high degree of versatility, allowing for straightforward modifications to adapt to various pathological conditions. The antimicrobial mechanism of CQDs is multifaceted and includes several pathways, as described below (Figure 5).

**FIGURE 5:**
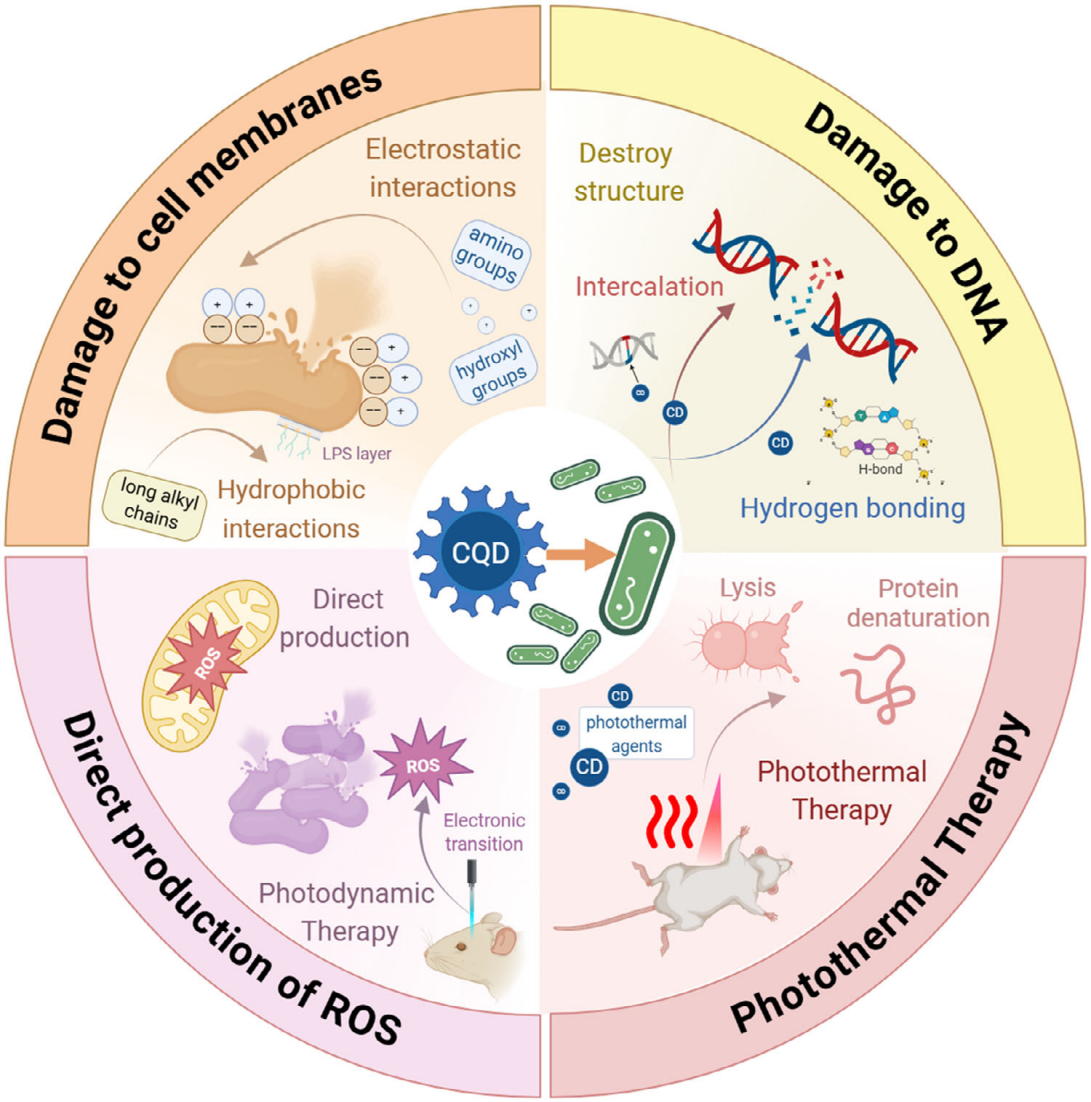
**Bactericidal and antibiofilm actions of CQDs**. Schematic illustration of the antibacterial mechanisms of CQDs, including interactions with the bacterial cell surface, DNA damage, direct reactive oxygen species generation, and photothermal therapy.

Damage to the cell membrane: CQDs can directly disrupt bacterial cell membranes through electrostatic and hydrophobic interactions. Positively charged CQDs interact electrostatically with the negatively charged surfaces of bacteria, facilitating their accumulation on the cell membrane and leading to bacterial rupture and death. Research indicates that NP‐CQD, which is rich in hydroxyl and amino groups, carries a positive charge and electrostatically binds with anions on the phospholipid bilayer of the cell membrane, damaging the bacterial surface and exhibiting significant bactericidal effects [143]. Similarly, positively charged CQDs, such as PC‐CQD, SpeY‐CQD, and N‐CQD, can anchor to and disrupt cell membranes through electrostatic attraction, demonstrating strong antibacterial effects [144, 145, 146].

Moreover, alkyl chains exhibit hydrophobic properties, and CQDs with long alkyl chains are more likely to penetrate the hydrophobic lipid layer of the cell membrane, thereby accelerating bacterial lysis. The alkyl chains on the surface of TAAC9‐CQD create a hydrophobic effect with the lipopolysaccharide layer, promoting the aggregation of CQDs on the cell membrane and thereby facilitating membrane rupture [147].

##### 4.2.1.1 Damage to DNA

CQDs can also induce irreparable DNA damage in bacteria. They primarily bind to DNA through mechanisms such as intercalation, electrostatic interactions, hydrophobic interactions, and hydrogen bonding [148, 149]. CQDs can disrupt the supercoiled structure of bacterial DNA, cleaving it into fragments and leading to bacterial death, thereby exerting their antimicrobial effects [150, 151]. Electrophoresis data indicate that DNA bands of bacteria treated with TPP‐CQDs significantly weaken or even disappear, suggesting that TPP‐CQDs can inflict substantial damage to bacterial DNA, leading to bacterial eradication [152].

In addition to directly killing bacterial cells, CQDs can also disrupt biofilms by damaging extracellular DNA. As a crucial component of biofilms, extracellular DNA interacts closely with extracellular polysaccharides and proteins, promoting biofilm formation and maturation. It helps maintain biofilm structure and acts as a signaling molecule to enhance communication among bacteria within the biofilm [153, 154]. Experimental findings indicate that Fe‐CQDs can directly act on biological membranes, resulting in membrane damage and a decrease in cellular activity; DNA fragments that have been cleaved can be extracted after the action of CQDs [155].

##### 4.2.1.2 ROS Generation

CQDs can directly generate ROS for antibacterial effects and function synergistically with PDT. Under light stimulation, CQDs undergo electronic transitions that produce ROS, thereby enhancing their antibacterial activity. When combined with PDT, CQDs rapidly absorb energy and convert it into an excited state, which can then facilitate electron transfer with intracellular substrates or directly with oxygen molecules to produce ROS [156, 157]. This process disrupts bacterial structures and biofilms.

The ROS content in bacteria significantly increases under the influence of Lys‐CQDs, affecting the normal physiological activities of bacteria. This increase leads to the decomposition of extracellular polymeric substances, resulting in a significant inhibition of both planktonic and biofilm states of the bacteria. Moreover, the mature biofilm structure is destroyed [158]. Similarly, OR‐CQD exhibits antimicrobial properties by elevating the concentration of ROS within bacteria, thereby damaging proteins and other cellular structures [159]. Additionally, F‐CQD and Cu‐CQD can also directly induce a notable increase in intracellular ROS in microorganisms, contributing to their bactericidal effects [160, 161].

Upon photoactivation, CE‐CQD generates O2•−, disrupting the intracellular ROS balance and inducing bacterial death. This process aids in the healing of infected wounds and plays a crucial role in promoting tissue regeneration [162]. Furthermore, H‐CQD demonstrates significant antimicrobial efficacy under laser irradiation, with experiments showing that it can penetrate bacteria to increase ROS concentration, achieving an antibacterial efficiency of up to 100% [163].

##### 4.2.1.3 Photothermal Therapy

Under light stimulation, CQDs with photothermal conversion properties can function as photothermal agents, rapidly increasing temperature and causing protein denaturation and cell lysis, thereby demonstrating excellent antibacterial effects [164]. For instance, Fe‐CQD can quickly elevate the local temperature upon light exposure, effectively inhibiting or even killing *Staphylococcus aureus* and *Escherichia coli*. The combination of photothermal interactions and ROS produced by CQDs exhibits superior antimicrobial effects [102]. Similarly, Cur‐CQD displays good photothermal stability and can effectively kill bacteria through localized heating under irradiation, demonstrating enhanced antibacterial effects when synergized with PDT [165].

While CQDs show great potential in combating microbial infections, some studies indicate that they attach more effectively to Gram‐positive bacteria and have greater damaging effects on them compared with Gram‐negative bacteria, due to differences in cell wall structure. Gram‐positive bacteria possess a thick layer of peptidoglycan in their cell walls, which is loosely structured and facilitates the direct attachment of CQDs. In contrast, Gram‐negative bacteria have a dense outer membrane rich in lipopolysaccharides, phospholipids, and proteins, presenting a greater barrier to carbon dot attachment and damage. To enhance the antibacterial efficacy of CQDs against Gram‐negative bacteria, future efforts may focus on introducing hydrophobic chemical groups or utilizing hydrophobic precursors. This approach could improve hydrophobic interactions between CQDs and the lipid layer, thereby promoting the adsorption of CQDs onto Gram‐negative bacteria and consequently enhancing their antibacterial action, which is crucial for reducing infection occurrence and aiding in the regeneration of infected tissues.

While broad‐spectrum antibacterial CQDs effectively target various bacterial types, they also inhibit the activity of beneficial bacteria, potentially disrupting the microbial balance. In contrast, narrow‐spectrum antibacterial CQDs have a more targeted ability to kill specific bacteria without harming beneficial bacteria. However, long‐term use of narrow‐spectrum antibacterial CQDs can increase the risk of developing drug resistance in specific target bacteria. Therefore, it is essential to select the appropriate CQDs for antibacterial treatment based on the types and characteristics of different infections. For drug‐resistant strains, a single killing mechanism of CQDs may not suffice to meet clinical needs. The development of CQDs with multiple antimicrobial mechanisms to act synergistically holds promise as a key direction for advancing the next generation of antimicrobial CQDs.

However, it is particularly important to note that when ROS production exceeds the scavenging capacity of the antioxidant system, excessive ROS accumulation not only induces oxidative damage and reduces cell viability but also further aggravates inflammatory responses by promoting calcium ion influx [64]. This indicates that CQDs relying on ROS‐mediated antibacterial activity have a strict therapeutic window. Beyond this range, increasing ROS production no longer improves antibacterial efficiency but instead increases the risk of damage to surrounding healthy tissues through nonspecific oxidative stress. Currently, most studies evaluate biosafety mainly through ROS fluorescent probes and cell viability assays, but rarely investigate the spatial diffusion range, duration of ROS activity, or tolerance thresholds of different cell types within tissues. Therefore, it remains difficult to accurately define the safety window of CQD‐mediated PDT/PTT therapies. To promote the clinical translation of CQD‐based phototherapy, future studies should not only optimize conventional parameters, such as CQD concentration and irradiation conditions, but also establish quantitative safety evaluation systems based on ROS exposure levels and tissue tolerance. These efforts should clarify the dose‐dependent relationship between therapeutic efficacy and oxidative damage, ensuring effective antibacterial activity while minimizing oxidative injury to healthy tissues, thereby providing a more reliable basis for the safe clinical application of CQDs.

#### 4.2.2 Regulating the Immune Microenvironment

The immune microenvironment is a critical ecological factor influencing the outcome of tissue regeneration and repair. Immune cells provide an essential defense barrier against invading pathogens by recognizing and eliminating bacteria and infected cells. However, due to bacterial persistence, immune evasion mechanisms, and the immune status of the host, prolonged inflammation and delayed tissue repair often occur. Therefore, regulating the activity of local immune cells can enhance antibacterial defense, reduce excessive inflammation, and establish an immune microenvironment favorable for tissue regeneration and repair [166]. Neutrophils, as the most abundant immune cell population, constitute the first line of innate immune defense and can rapidly engulf invading pathogens. Macrophages act as scavengers of the immune system by removing cellular debris and pathogens while activating other immune responses. Meanwhile, the lymphatic system maintains immune microenvironmental homeostasis by regulating immune cell migration and facilitating the clearance of inflammatory mediators [167]. Specifically, CQDs can enhance the antibacterial activity of neutrophils by activating neutrophil‐mediated NETosis, thereby promoting bacterial capture and elimination. CQDs can also restore macrophage‐mediated efferocytosis, facilitate the timely clearance of apoptotic cells, and suppress excessive inflammatory responses by downregulating pro‐inflammatory genes and related signaling pathways (Figure 6).

**FIGURE 6:**
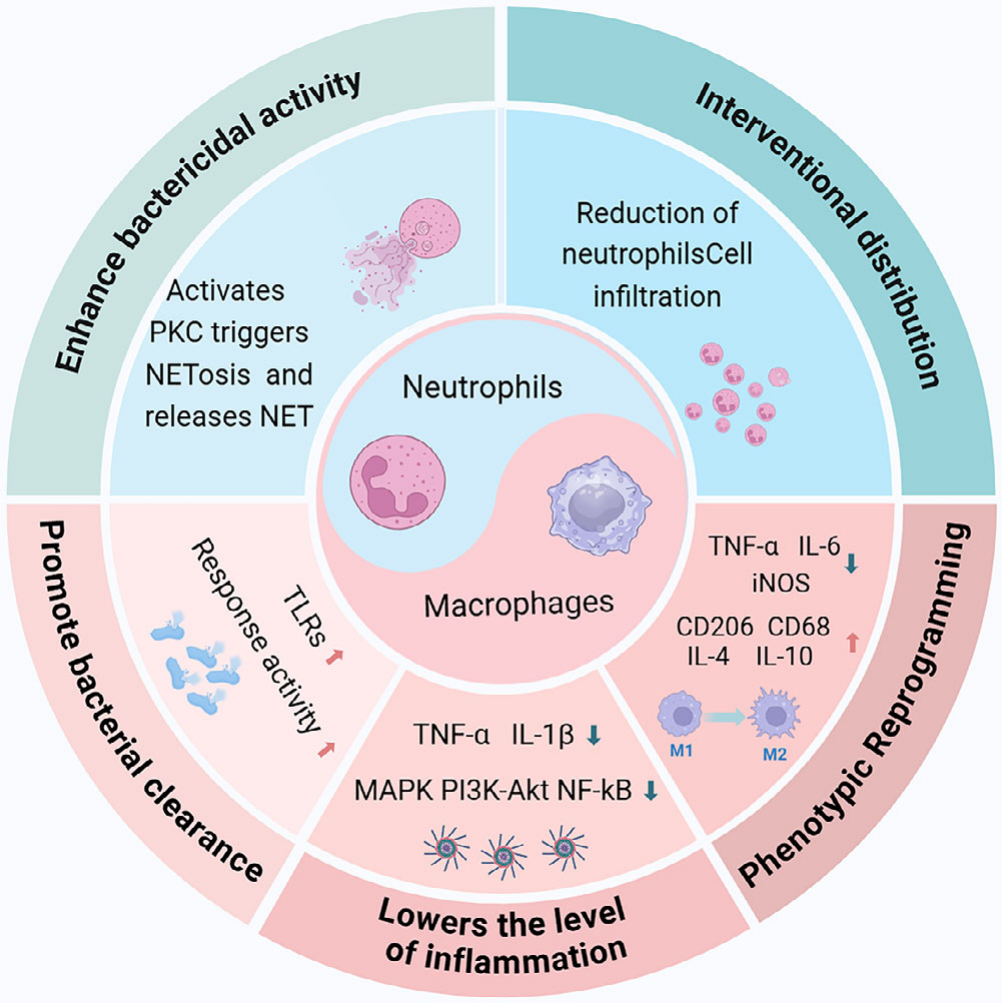
**Immune microenvironment regulation by CQDs**. Schematic illustration of the effects of CQDs on neutrophils and macrophages and their roles in regulating the inflammatory and immune microenvironment during tissue regeneration.

##### 4.2.2.1 Regulating Neutrophils

NETosis is a form of programmed cell death that occurs in neutrophils following specific stimuli, during which neutrophil extracellular traps (NETs) are released. NETs contain antimicrobial proteins and oxidases that can capture and kill pathogens, making them a crucial component of innate immunity [168]. CQDs, such as CCM‐CQD, can effectively activate NETosis and induce NET formation via a NOX‐dependent pathway. The activated neutrophils then kill bacteria efficiently by producing hypochlorous acid through myeloperoxidase catalysis. Additionally, the unique network structure of NETs allows them to physically adhere to bacteria, enhancing their antimicrobial effectiveness. Mechanistic studies have revealed that CCM‐CQD activates protein kinase C by stimulating phorbol 12‐myristate 13‐acetate, which in turn promotes NADPH oxidase synthesis and ROS production. This cascade activates neutrophils to release NETs, further contributing to their bacterial killing ability [169].

While inflammation is a natural response to bacterial infection, persistent inflammation can be detrimental to tissue regeneration. Chronic inflammation causes cell damage that hinders the recovery of regenerated tissues [170, 171]. The prolonged presence of neutrophils can exacerbate the local inflammatory response in tissues. Therefore, the removal of resident neutrophils through processes such as apoptosis can help alleviate the inflammatory state and promote tissue repair. For example, C‐GQD has been shown to reduce neutrophil infiltration near the wound, helping to restore immune homeostasis and promote fibroblast proliferation [172].

##### 4.2.2.2 Regulating Macrophages

Macrophage phenotypes can be classified into classically activated macrophages (M1), which possess strong bactericidal functions, and alternatively activated macrophages (M2), which are involved in anti‐inflammatory repair processes [173, 174]. CQDs can enhance antimicrobial activity by increasing the responsiveness of macrophages. For instance, PL‐CQD upregulates the expression of tumor necrosis factor (TNF) and NF‐κB, heightening macrophage sensitivity to IL‐1β and TNF, thereby strengthening bacterial defenses [175].

Notably, the role of inflammatory responses in tissue regeneration is dual‐edged; while mild inflammation in the early stages facilitates bacterial clearance and helps prevent infection, sustained inflammation increases the risk of nonhealing wounds. Studies have shown that various CQDs can downregulate the MAPK pathway, actively mitigating inflammation. The MAPK pathway plays a key regulatory role in the pro‐inflammatory functions of macrophages by promoting the expression of TNF, IL‐1β, and IL‐10, and sustained activation of this pathway can lead to excessive inflammation [176]. FA‐CQD specifically targets the MAPK pathway, blocking its activation, downregulating the expression of PI3K–AKT and NF‐κB, and reducing the excessive production of inflammatory cytokines by macrophages [176, 177].

Moreover, CQDs can promote the reprogramming of macrophages to their reparative phenotype. FA‐CQD has been shown to inhibit the expression of TNF and IL‐6 and downregulate inducible nitric oxide synthase levels, thereby remodeling the macrophage phenotype and allowing M1 macrophages to transform into M2 macrophages [177]. Other CQDs can induce macrophage polarization toward the M2 phenotype through various mechanisms; for example, C‐CQD activates the TGF‐β–SMAD pathway by inhibiting *Smad7* expression, Ag‐CQD upregulates the expression of CD206 and CD68, while FCQD enhances the expression of IL‐4 and IL‐10 [103, 178, 179, 180]. These synergistic effects help reduce inflammation and improve the overall outcomes of wound healing and bone regeneration. T‐CQD, in particular, can promote the transformation of macrophages to an anti‐inflammatory repair phenotype in diabetic wounds, facilitating their healing [181].

Regulation of efferocytosis and lymphangiogenesis: In recent years, macrophage‐mediated efferocytosis has attracted increasing attention as an important immune regulatory mechanism connecting apoptotic cell clearance, inflammation resolution, and tissue regeneration. Specifically, Mn‐CQD can reshape the gut microbiota to increase the production of indole‐3‐propionic acid, which subsequently upregulates the expression of the efferocytosis receptor MERTK and enhances efferocytosis, thereby driving macrophage polarization toward the anti‐inflammatory M2 phenotype [182]. However, this effect is indirectly mediated through the gut microbiota and its metabolites. Whether CQDs can directly target efferocytosis‐related pathways remains largely unexplored and requires further investigation. In addition to efferocytosis, the important role of the lymphatic system in regulating the immune microenvironment has become increasingly recognized. A study investigating vascular regeneration found that Cb‐CQD could promote the expression of β‐catenin, a key factor associated with the Wnt signaling pathway [183]. Notably, activation of the WNT signaling pathway is also involved in lymphangiogenesis and can promote lymphatic regeneration by regulating lymphatic endothelial cell functions [184]. However, direct evidence demonstrating whether CQDs can specifically promote lymphatic regeneration is currently lacking, and further studies at both cellular and animal levels are needed to establish clear causal relationships.

Furthermore, various immune cell populations, including regulatory T cells (Tregs), innate lymphoid cells (ILCs), γδ T cells, and eosinophils, play important roles in maintaining immune homeostasis, coordinating tissue regeneration, and promoting repair processes. However, the regulatory effects and underlying mechanisms of CQDs on these immune cells have rarely been reported. The intrinsic biological activities of CQDs may provide substantial potential and therapeutic opportunities for modulating these immune populations. Meanwhile, accumulating studies have gradually revealed the complexity of immune regulation. The efferocytosis functions of professional phagocytes, such as macrophages and dendritic cells, play essential roles in maintaining tissue homeostasis and promoting repair and regeneration, while potential regulatory targets involved in these processes continue to be identified. Similarly, regulatory mechanisms of lymphatic regeneration, as a critical component of tissue repair and immune surveillance, have received increasing attention. Based on these advances, the development of novel CQDs designed to regulate efferocytosis or promote lymphangiogenesis as therapeutic strategies represents a promising direction for future research.

#### 4.2.3 Removal of ROS

ROS play distinct roles at various stages of tissue regeneration and wound healing. Moderate levels of ROS can induce mild inflammation in the early stages, effectively recruiting immune cells and exerting bactericidal effects. However, excessive ROS can prolong inflammation, damage organelles and cells, and hinder tissue regeneration, making their removal essential for optimal healing.

Current research indicates that CQDs primarily act as electron donors through specific functional groups, engaging in redox reactions with free radicals [185]. By donating electrons, CQDs can neutralize harmful ROS, thus contributing to a reduction in oxidative stress. Additionally, these nanomaterials can indirectly enhance the activity of antioxidant enzymes, aiding in the elimination of ROS by regulating various cellular pathways (Figure 7).

**FIGURE 7:**
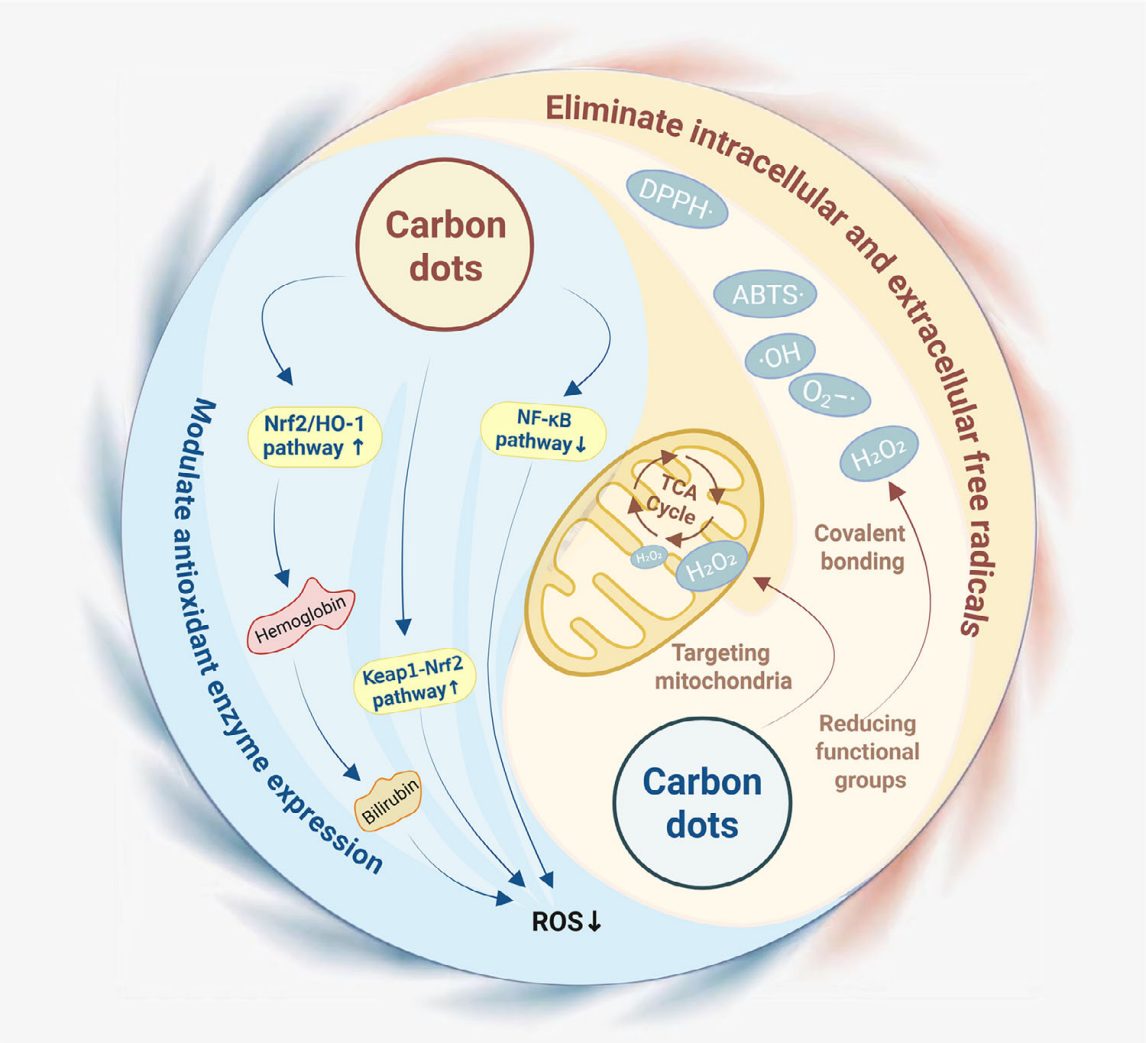
**Removal of reactive oxygen species by CQDs**. Schematic illustration of the direct and indirect mechanisms by which CQDs regulate reactive oxygen species levels through direct scavenging and modulation of endogenous antioxidant responses.

Elimination of extracellular free radicals: Due to the abundant reducing functional groups, such as ketone carbonyl, hydroxyl, and carboxyl groups on the surface, CQDs can directly remove the excess ·OH, O2•−, DPPH·, and H_2_O_2_ [185, 186]. In vitro experiments showed that ZIF‐Cu‐CQD could eliminate O2•− and ·OH to reduce ROS levels and has an antioxidative stress effect [63].

Elimination of intracellular free radicals: Intracellularly, CQDs can selectively scavenge free radicals; for example, HOCQD and DACQD are effective at scavenging ·OH, and GSH‐CQD and NAC‐CQD are effective at scavenging O2•− [187, 188, 189]. Experiments have demonstrated that AA‐CQD can effectively scavenge various reactive species in cells, including 2,2'‐azinobis(3‐ethylbenzothiazoline‐6‐sulfonic acid) radical, DPPH·, ·OH, and O2•−, with the highest efficacy observed in the elimination of ·OH. Further research indicates that AA‐CQD removes ROS through covalent bonding, such as through the formation of ether bonds [190]. PB‐CQD can eliminate ·OH and specifically target mitochondria within cells by acting directly on the primary sites of ROS production, effectively removing H_2_O_2_ from the mitochondria and thereby reducing mitochondrial damage [191].

Enhancing the antioxidant capacity of cells: The Nrf2–HO‐1 pathway plays a crucial role in eliminating ROS and protecting cells.  NRF2 induces the expression of HO‐1, which degrades heme to form bilirubin under oxidative stress, thereby reducing ROS levels and enhancing the antioxidant capacity of cells [192, 193]. MT‐CQD activates the NRF2–HO‐1 pathway, increasing the expression of  NRF2 and HO‐1 proteins, which effectively clears ROS, combats inflammation, and reduces cell damage [194]. Additionally, KEAP1 acts as an endogenous inhibitor of NRF2, and NAC‐CPD activates the KEAP1–NRF2 pathway, reducing intracellular ROS and lowering inflammation levels [195, 196].

CQDs not only enhance the expression of antioxidant enzymes but also help prevent the activation of ROS during inflammation. The activation of the NF‐κB signaling pathway is often associated with the onset of inflammatory responses. Studies have indicated that CQDs can suppress the NF‐κB signaling pathway and mitigate oxidative stress by decreasing the expression of P65 [186, 197, 198]. Another study has shown that NAC‐CPD can inhibit the NF‐κB pathway by downregulating the expression of Toll‐like receptor 4 (TLR4) [196]. Once ROS are produced, the resulting damage can be irreversible, making early intervention crucial in preventing the onset and progression of diseases [75, 199]. Previous studies suggest that CQDs can enter and distribute within mitochondria, targeting these essential organelles that are crucial for cellular energy metabolism. The electron transfer process in the mitochondrial respiratory chain serves as a significant pathway for ROS production. Owing to their mitochondrial‐targeting ability, CQDs accumulate precisely at the primary sites of ROS generation, thereby enhancing their capacity to neutralize free radicals before oxidative damage propagates.

Therefore, targeting the pathways of ROS production in mitochondria is expected to provide new insights into potential applications for CQDs. Additionally, surface chemical modifications of CQDs or the incorporation of reducing functional groups could be strategically implemented to enhance their antioxidant properties, further augmenting their capacity to scavenge ROS.

CQDs can indirectly promote tissue regeneration by targeting multiple pathological processes, including infection, inflammation, and oxidative stress, thereby improving the microenvironment of damaged tissues. This feature is particularly important for tissue injuries with complex pathological microenvironments, such as chronic wounds. Building on these properties, smart wound dressings that integrate the stimulus‐responsive and multifunctional characteristics of CQDs could further combine antibacterial activity, immune regulation, and redox control [64]. By dynamically monitoring wound status, these systems may enable more localized and controllable treatment, providing a new direction for the application of CQDs in tissue regeneration. However, increasing functional complexity also makes dose control, manufacturing consistency, and safety evaluation more challenging. Whether these multifunctional systems can ultimately achieve clinical translation remains to be determined.

## 5 Preclinical Research and Clinical Translation of CQDs

CQDs have demonstrated promising potential in precision therapy and tissue repair, and increasing efforts have been devoted to understanding their underlying mechanisms. However, their overall clinical translation remains at an early stage. Currently, many preclinical studies rely on in vitro cellular models to evaluate the therapeutic effects of CQDs and their ability to promote soft and hard tissue repair and regeneration [200, 201]. For animal model validation, existing studies mainly use mouse or rat models, while systematic evaluation in large animal models and long‐term efficacy studies remain limited. These limitations make it difficult to fully reproduce the complex microenvironment of the human body [202]. At present, most safety evaluations of CQDs focus on short‐term cytotoxicity assessments, while their long‐term metabolism, biodistribution, immune responses, and potential risks in vivo remain insufficiently understood. Meanwhile, differences in CQD types, dosages, and evaluation criteria among different studies make direct comparison of results difficult. In addition, limited control over synthesis processes, lack of standardized characterization methods, and incomplete regulatory frameworks further create substantial barriers to the clinical translation of CQDs.

Compared with traditional biomaterials, CQDs represent a class of emerging nanomaterials with complex compositions and diverse structures. Their long‐term biosafety, in vivo behaviors, and quality control systems have not yet been systematically evaluated. Therefore, advancing CQDs from experimental research toward clinical applications requires the establishment of a complete translation pathway based on pharmaceutical development standards, including standardized preparation and comprehensive evaluation systems. During this process, the primary challenge in transforming CQDs from experimental materials into standardized products is the establishment of standardized synthesis and structural characterization approaches. Since the properties of CQDs are highly dependent on precursor sources, synthesis conditions, and posttreatment processes, CQDs prepared using the same method may still exhibit differences in particle size, surface functional groups, and electronic structures among different batches. These variations not only affect the biological effects of CQDs but also increase uncertainty in mechanistic studies and clinical evaluations. Therefore, future studies should establish standardized preparation systems that meet clinical requirements, identify key structural parameters, and optimize reproducible synthesis processes. In addition, deeper investigation into the relationship between structural characteristics and therapeutic effects is needed to facilitate the transition from empirical screening toward rational design.

Beyond preparation standardization, systematic safety evaluation represents another major factor limiting the clinical translation of CQDs. Before entering clinical trials, comprehensive preclinical evaluation systems should be established to systematically characterize the toxicological properties, in vivo behaviors, and immune‐related risks of CQDs. These evaluations should include acute and chronic toxicity studies, pharmacokinetic analyses, biodistribution investigations, and immunogenicity assessments, thereby providing reliable safety evidence for clinical applications. Moreover, because CQDs exert therapeutic effects through regulation of ROS levels and immune responses, particular attention should be paid to maintaining the balance between therapeutic dosage and biological effects. This approach can help avoid adverse effects caused by excessive oxidative stress or abnormal immune activation. Future studies should establish standardized animal models and unified evaluation systems to further define the effective dose ranges, duration of action, and long‐term safety profiles of different CQD types, providing reliable guidance for dose exploration in subsequent phase I clinical trials.

Taken together, CQDs have accumulated substantial preclinical research evidence, but a considerable gap remains before their clinical application. Future development of CQDs should not only focus on discovering new biological effects but also prioritize the establishment of therapeutic systems with stable quality, controllable safety, and clearly defined mechanisms of action. These efforts will facilitate the transition of CQDs from laboratory research toward clinical applications.

## 6 Conclusions and Perspectives

The high biological activity of CQDs and their ability to overcome multiple biological barriers enable them to play important roles not only in precision therapy but also as promising candidates for initiating deep tissue repair. Compared with traditional approaches, CQDs can improve biological utilization, penetrate deep tissues, reshape the tissue regenerative niche, and activate intrinsic self‐repair mechanisms. This review summarizes the biomedical applications of CQDs and particularly highlights their roles and molecular mechanisms in regulating tissue regeneration. However, several challenges remain that may limit their clinical application. Therefore, this review objectively analyzes the current challenges associated with CQD applications, explores their underlying causes, and proposes potential strategies to provide valuable references for researchers and clinicians.

### 6.1 Microstructure

To date, the relationship between the structure and function of CQDs has been gradually explored, but their precise molecular structures remain incompletely understood. Unlike traditional small‐molecule materials with well‐defined chemical structures, CQDs are generally composed of diverse carbon core structures, defect states, and complex surface functional groups. This complexity may result from diverse and unpredictable changes in precursor materials and reaction conditions during synthesis, making accurate structural identification difficult. In addition, the intrinsic amorphous characteristics of some CQDs further complicate their structural analysis [200]. Insufficient structural characterization of CQDs is one of the major reasons for the differences among current research findings. Even when similar synthesis methods are applied, CQDs produced by different laboratories may exhibit substantial variations in particle size distribution, fluorescence properties, ROS generation capacity, cellular uptake efficiency, and immunomodulatory effects. These differences are not solely caused by experimental conditions but are also closely associated with variations in Internal structures of CQDs, surface functional group density, heteroatom doping ratios, and the proportion of sp^2^/sp^3^ carbon structures. Therefore, establishing high‐resolution and multidimensional structural characterization systems is essential for accurately correlating CQD structures with their biological functions.

Future studies should combine advanced structural characterization techniques to further elucidate the microstructural composition of CQDs. In addition, machine learning and artificial intelligence (AI)‐based approaches can be applied to reveal the precise molecular structures of CQDs by integrating multidimensional characterization data. These approaches may help establish predictive models linking CQDs' structural features with biological effects, improve the predictability of CQD functions, and guide the rational design of new CQDs with specific biological activities.

### 6.2 Immune Regulation and Lymphatic Regeneration

Due to the inherent immunogenicity and immune escape capabilities of CQDs, further studies are necessary to clarify whether they have adverse effects on the internal environment and to assess their potential long‐term toxicity before clinical application. In order to mitigate excessive stimulation of the immune system, CQDs can be formulated as sustained‐release drugs, allowing for controlled release rates within the body and enhancing their targeted delivery effect. This approach can significantly improve the controllability and precision of carbon dot release in tissues.

Additionally, the lymphatic system functions as a multifunctional transport network that integrates immune regulation, the maintenance of homeostasis, and the clearance of metabolic products. It plays a critical role in maintaining a balance between immune recognition and antigen presentation, providing a conducive microenvironment for tissue regeneration [200, 203]. Therefore, promoting lymphatic vessel regeneration presents an effective strategy for regulating the immune response and stimulating tissue repair in cases of damage or defect. However, the mechanisms of interaction between CQDs and lymphatic endothelial cells remain underexplored. Further theoretical and experimental studies are needed to elucidate the effects of CQDs on the lymphatic system.

### 6.3 Multimechanism Collaboration

To enhance the efficacy and efficiency of CQDs in the induction of tissue regeneration, surface modification techniques can be employed to make them multifunctional, thus presenting various mechanisms of action to promote tissue regeneration through multiple pathways. Additionally, CQDs can be combined with other biomaterials to exhibit synergistic effects, such as bone scaffold materials or 3D‐printed bioinks. Such combinations allow the materials to complement each other, resulting in a more comprehensive regenerative effect.

### 6.4 Commercial Production

To facilitate the smooth transition of CQDs from the laboratory to clinical application, it is essential to optimize their manufacturing technology to ensure the industrial production of a large quantity of uniform‐sized, high‐performance CQDs; this could be achieved through the precise control of reaction conditions, thereby improving production efficiency. Leveraging the high bioactivity and exceptional delivery efficiency of CQDs to initiate the cascade of tissue regeneration offers significant advantages in terms of treatment efficacy and anatomical accuracy. Understanding the functional processes and molecular mechanisms of CQDs is crucial to enhance their clinical translation and provide valuable insights for the design of future carbon dot formulations. Through multidisciplinary collaboration, we can expand the range of applications for CQDs, ultimately providing more personalized and precise approaches for clinical treatments. This collaborative effort will be vital in unlocking the full potential of CQDs in regenerative medicine and improving patient outcomes.

## Author Contributions

Bingxin Fan, Nianqiang Jin, Nuo Jin: Writing the manuscript. Jiayu Shen, Xinliang Duan, Sichen Han, Yao Yuan: Validation. Zilin Wang: Project administration and writing the manuscript. All the authors have approved the final manuscript.

## Funding

This study was funded by the National Natural Science Foundation of China (Grant Nos. 82301026, 82503027); the Jilin Provincial Health Commission's Health Science and Technology Capacity Enhancement Program (Grant No. 2026WS‐YA023); the Graduate Innovation Fund of Jilin University (Grant Nos. 2026CX332, 2026CX338, 2026CX343); the College Students' Innovation and Entrepreneurship Training Program (Grant Nos. S202610183765, X202610183490).

## Ethics Statement

The authors have nothing to report.

## Conflicts of Interest

The authors declare no conflicts of interest.

## Data Availability

The authors have nothing to report.
